# Supplementary figures and images for: Cell type-agnostic transcriptomic signatures enable uniform comparisons of neural maturation
Source: PLoS Biol. 2026 Apr 15;24(4):e3003757. doi: 10.1371/journal.pbio.3003757 (PMC13095120; doi:10.1371/journal.pbio.3003757)

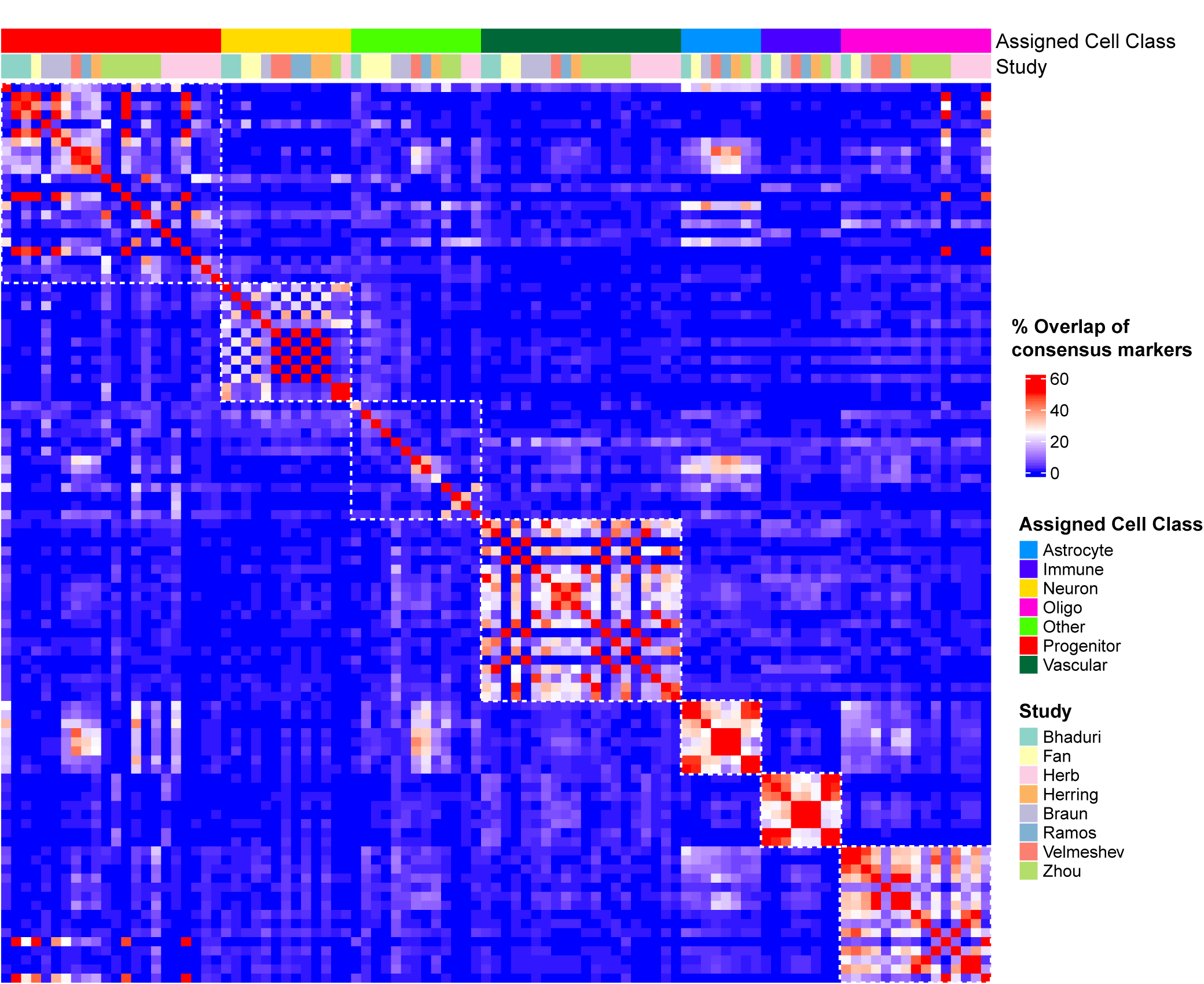

Supplement: S1 Fig — Author-provided cell type annotations from each study were grouped into 7 broad cell types (Astrocyte, Progenitor, Neuron, Oligodendrocyte, Immune, Vascular, and Others; see S2 Table). Heatmap shows the overlap of top 100 consensus marker genes for each cell type across datasets. Cell classes show consistent overlaps in consensus markers across datasets, except for “Other” cells which include multiple different cell types. (TIF) [file pbio.3003757.s001.tif]

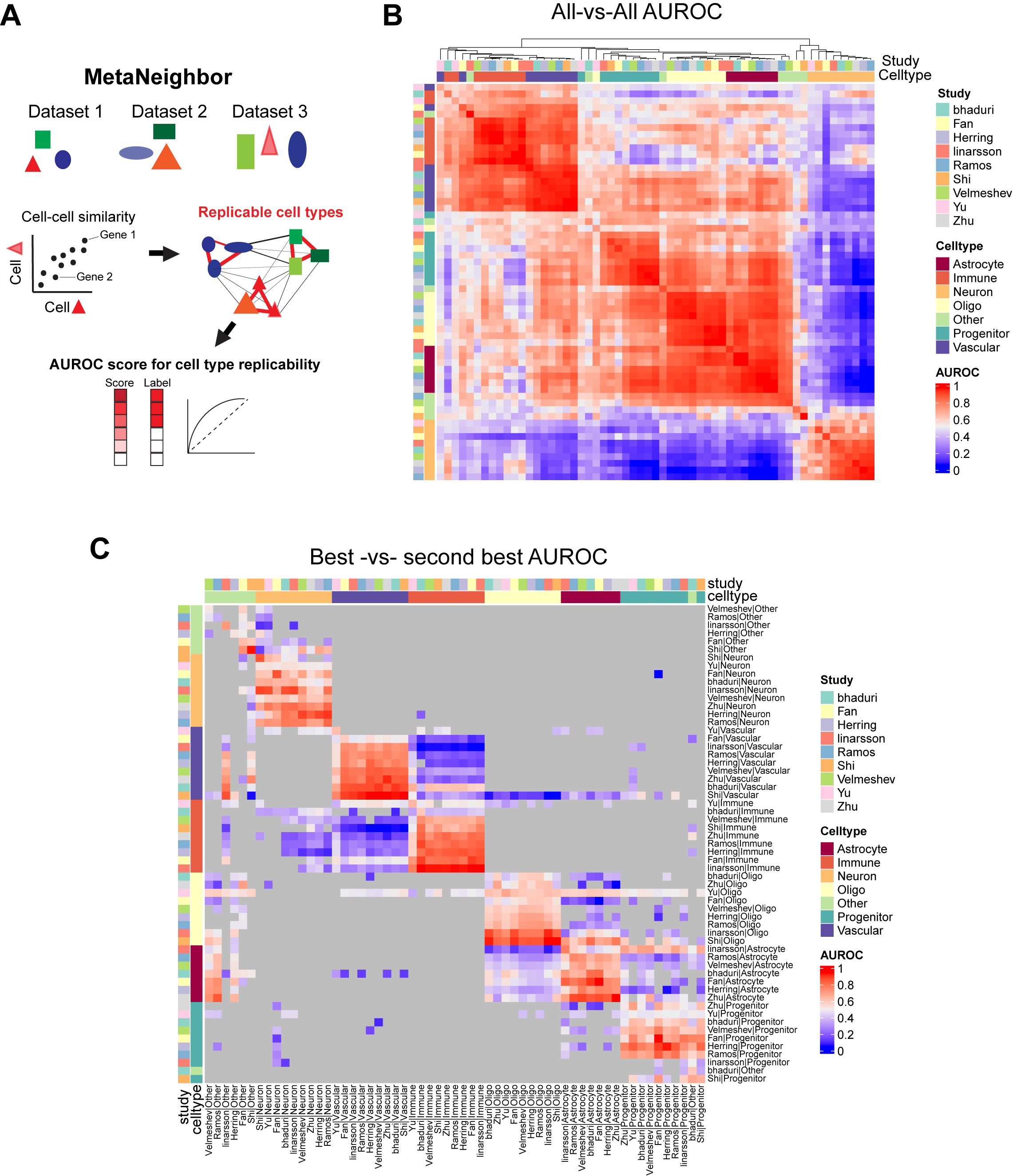

Supplement: S2 Fig — A, Schematic illustrating MetaNeighbor procedure: cell similarity networks constructed from gene-gene correlation are used to assess cell type replicability with a leave-study out cross-validation approach. The AUROC score measures whether cells of the same type rank higher than other cell 4 types in the network. B, Heatmap shows MetaNeighbor AUROC scores for all versus all comparisons of major cell classes across 9 datasets used for model training. Cells cluster by cell type rather than study C, Heatmap shows best versus next best MetaNeighbor AUROC scores measuring the ability to discriminate cells of the same type from the next closest cell type. Cells cluster by cell type rather than study. (TIF) [file pbio.3003757.s002.tif]

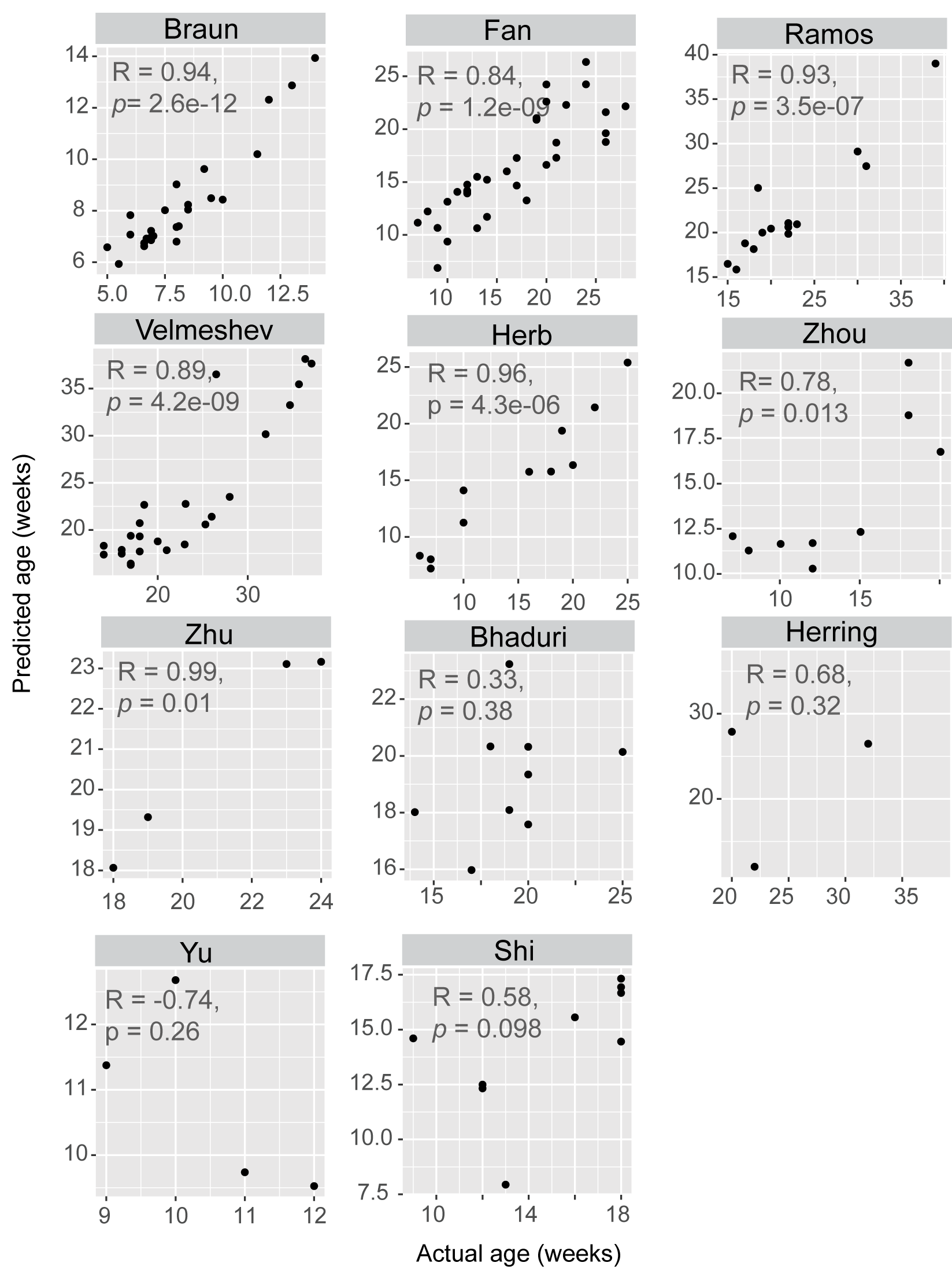

Supplement: S3 Fig — Performance of regularized regression models trained to predict sample age from cell class proportions within each study. Study-specific compositional models accurately predict gestational age in 7 out of 11 studies, with significant correlation between predicted and actual ages. (TIF) [file pbio.3003757.s003.tif]

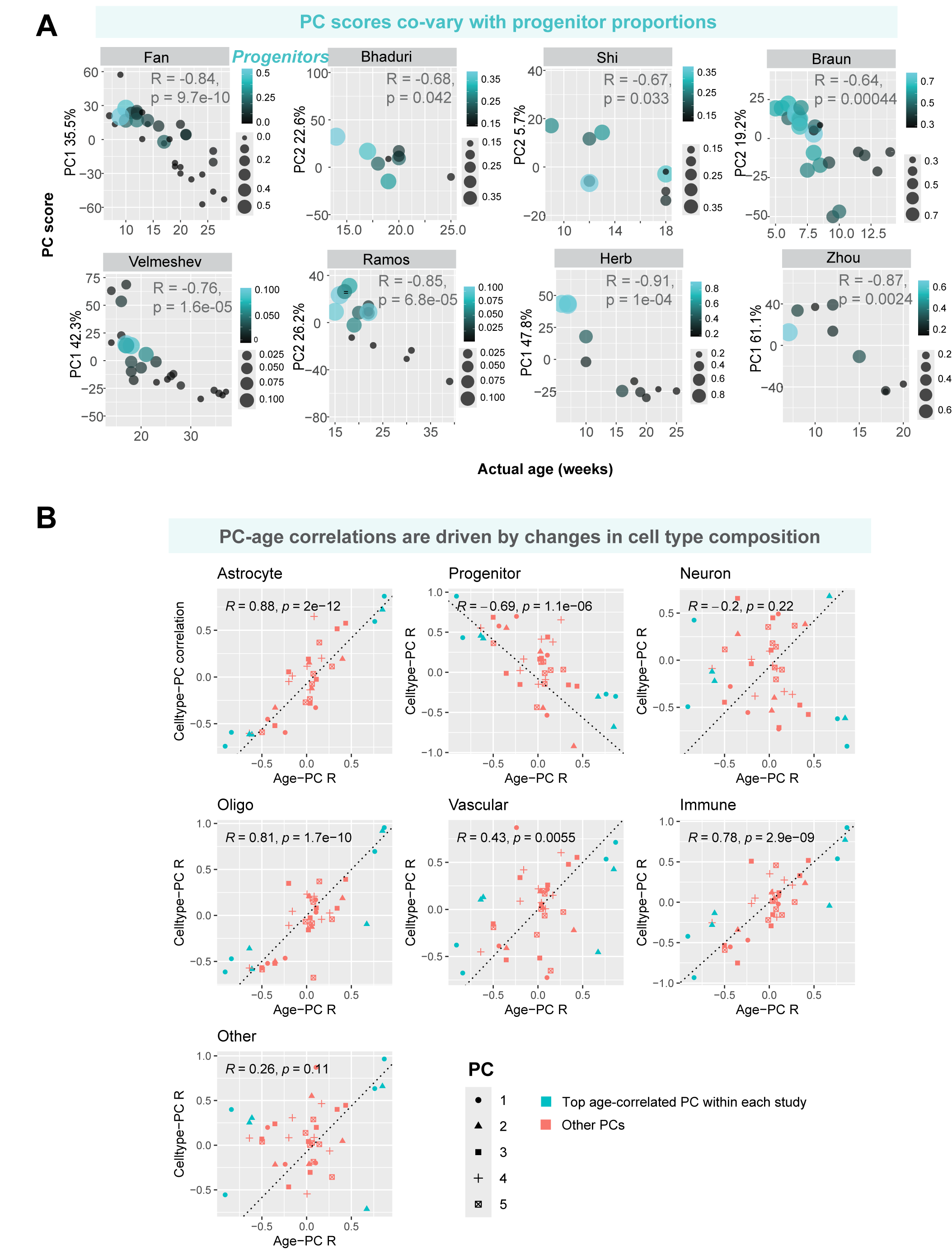

Supplement: S4 Fig — A, PC1 or PC2 scores are strongly correlated to age within each of the 8 datasets. Size of dots represents the proportion of progenitor cells in the sample. Percentage of variance explained by the PC is shown on the y-axis label. B, Correlation of PC with age is plotted against correlation of the PC with proportions for each cell type. Age-PC correlations are perfectly matched by PC-cell type proportion correlations for most cell types across all datasets. Shape of points indicates which principal component, and color represents the PC that was used for age prediction in panel A. (TIF) [file pbio.3003757.s004.tif]

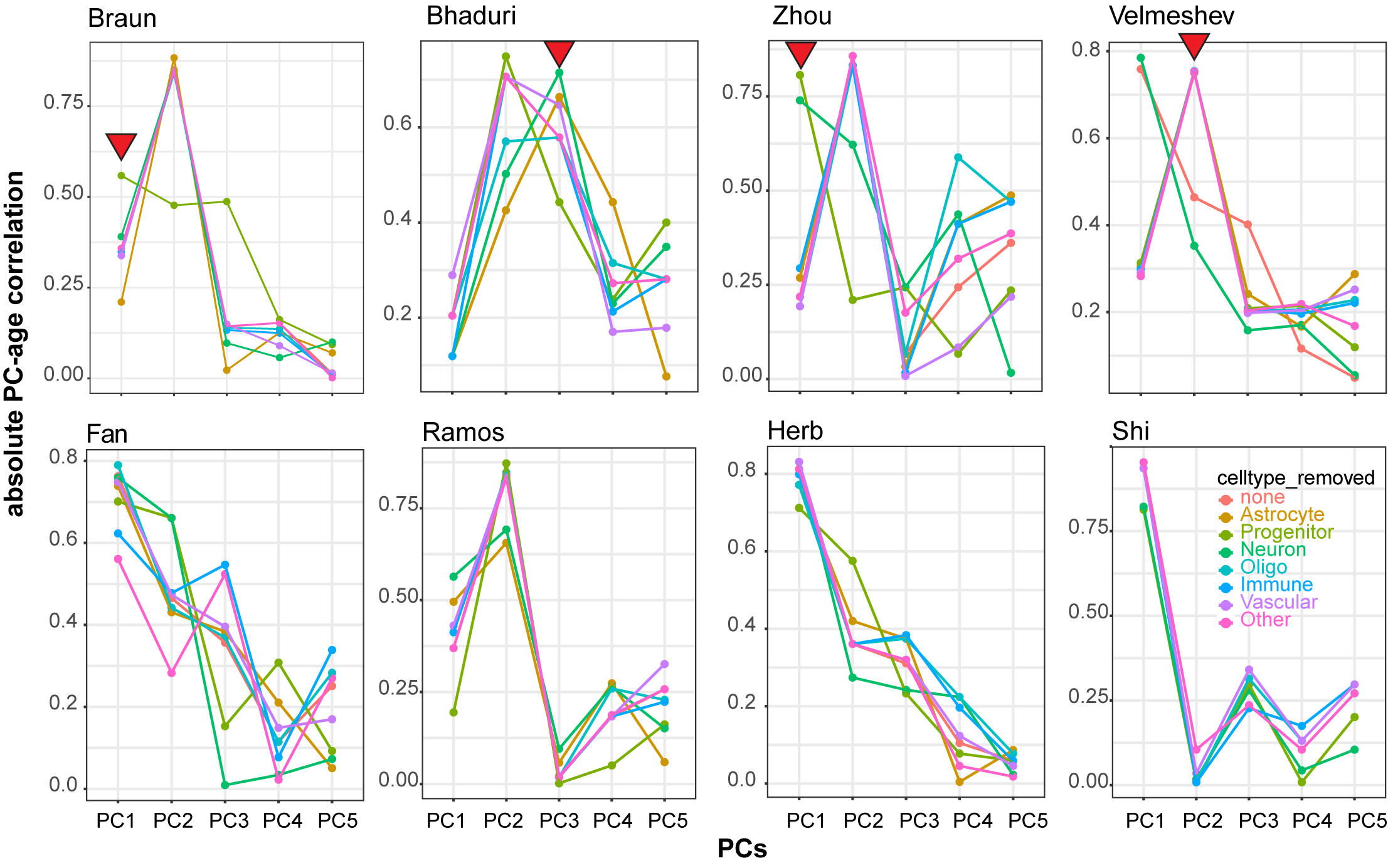

Supplement: S5 Fig — Panels show the absolute correlation coefficient between principal components (PC1–PC5) and age in each dataset. PCs were computed from pseudo-bulked gene expression. Colors indicate PCs recomputed after removing all cells of a specific cell type from the dataset. Removing cell types shifts the PC-age-correlation in 4/8 datasets shown in the top row. Red arrows indicate complete change in the PC-age-correlation: i.e., in Braun and colleagues, removing Progenitors from the dataset shifts the age-correlation from PC2 to PC1. In Zhou and colleagues, removing Progenitors or Neurons from the dataset shifts age-correlation from PC2 to PC1. (TIF) [file pbio.3003757.s005.tif]

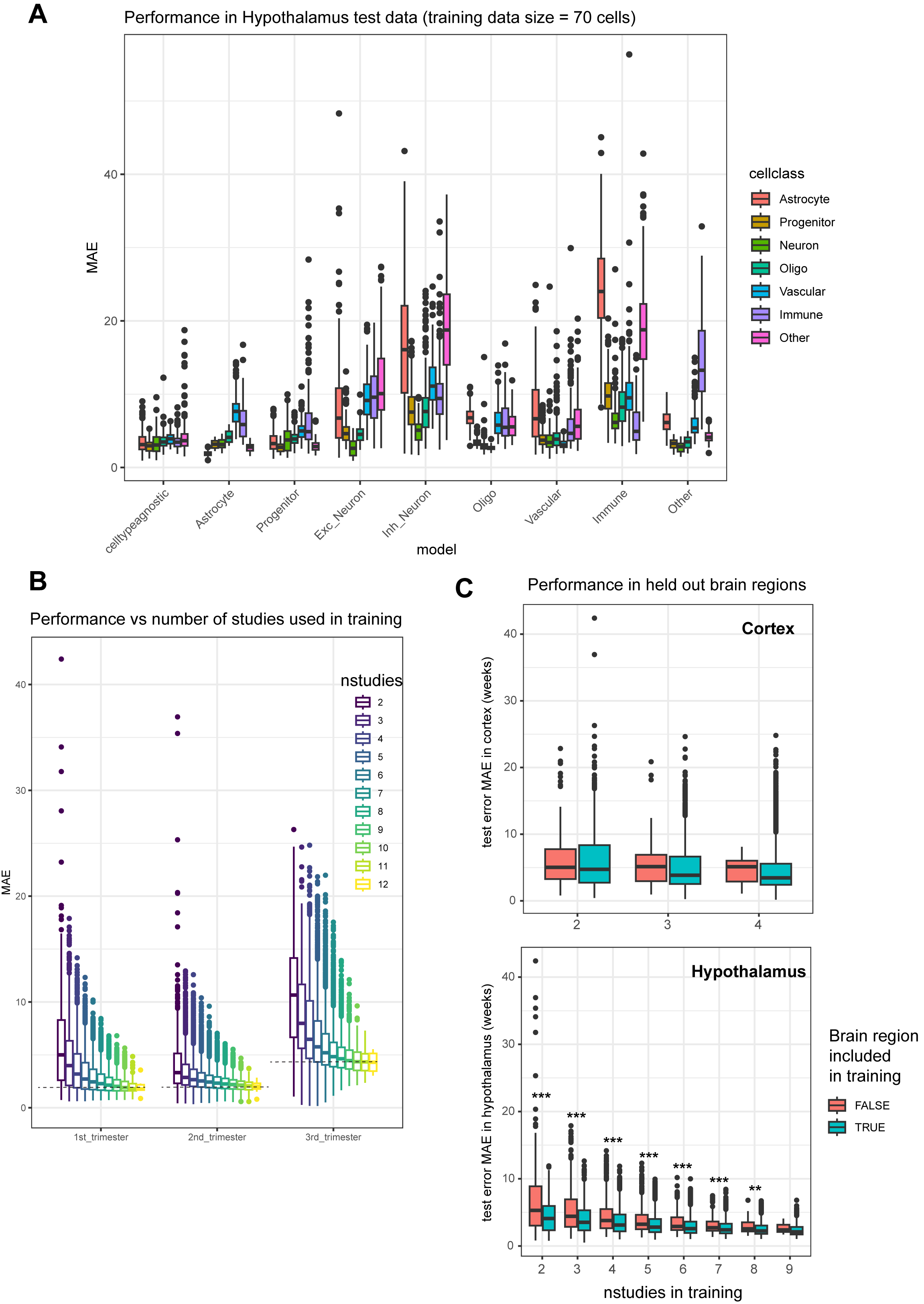

Supplement: S6 Fig — A, Boxplots show distribution of prediction error (Mean Absolute Error, MAE) in test data for 100 cell type-specific and cell type-agnostic models that were trained on the same number of cells (n = 70). B, Distribution of prediction error for models trained on different combinations of studies (N = 2–12). Performance increases as more datasets are used for training, but plateaus after ~7 studies. Third-trimester samples show highest prediction error due to scarcity of training data. C, Distribution of prediction error in test datasets from cortex (top) or hypothalamus (bottom) when these brain regions are included or excluded from training data. ***P < 0.001, **P < 0.01 (FDR-adjusted Wilcoxon p-value) (TIF) [file pbio.3003757.s006.tif]

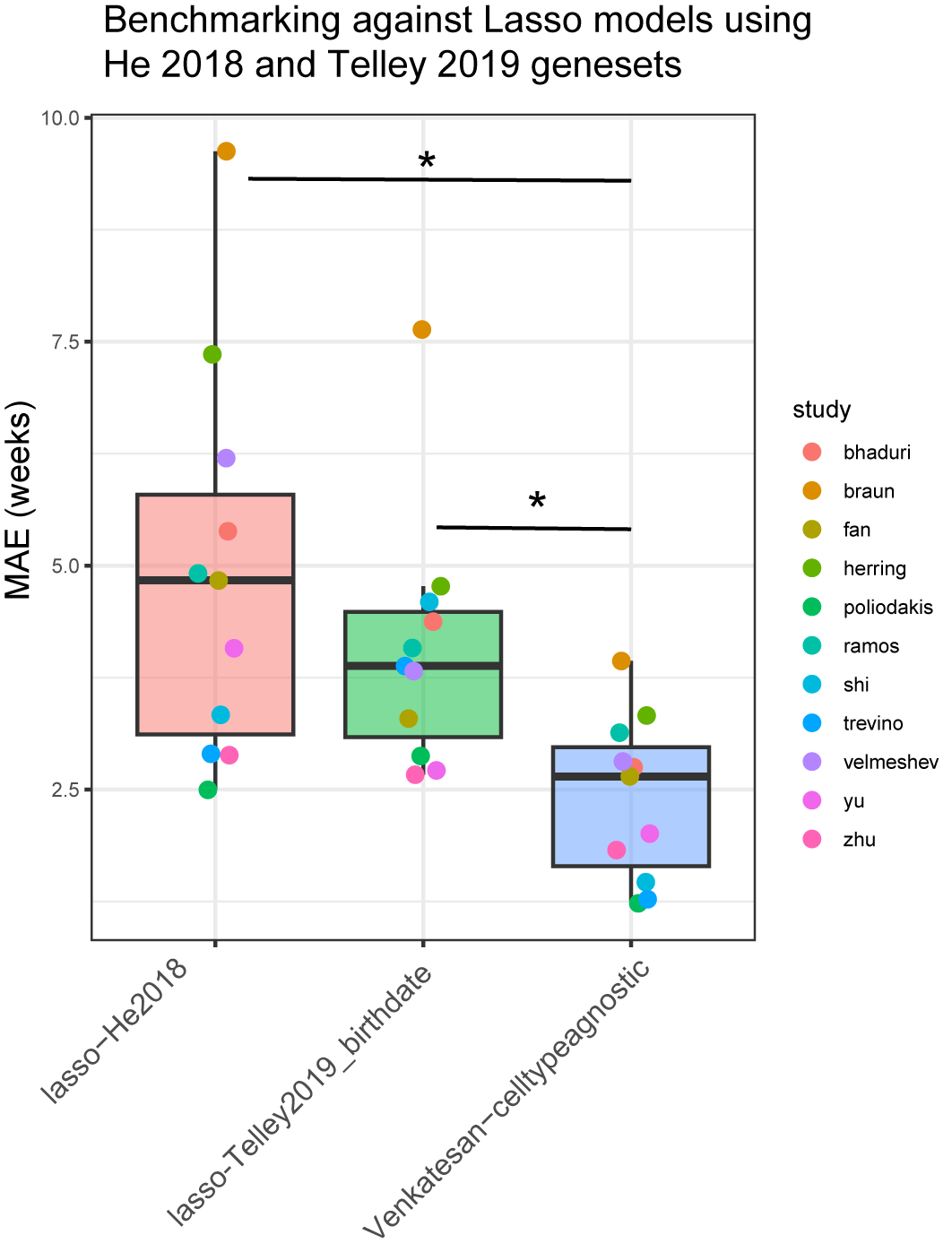

Supplement: S7 Fig — L1 (LASSO) regularized regression models to predict age were trained using neuronal maturation gene sets from He and colleagues 2018 and Telley and colleagues 2019. Boxplots compare prediction accuracy (mean absolute error, MAE) in each of the human fetal datasets to the cell type-agnostic model developed in this study. (* adjusted Wilcoxon P < 0.05). (TIF) [file pbio.3003757.s007.tif]

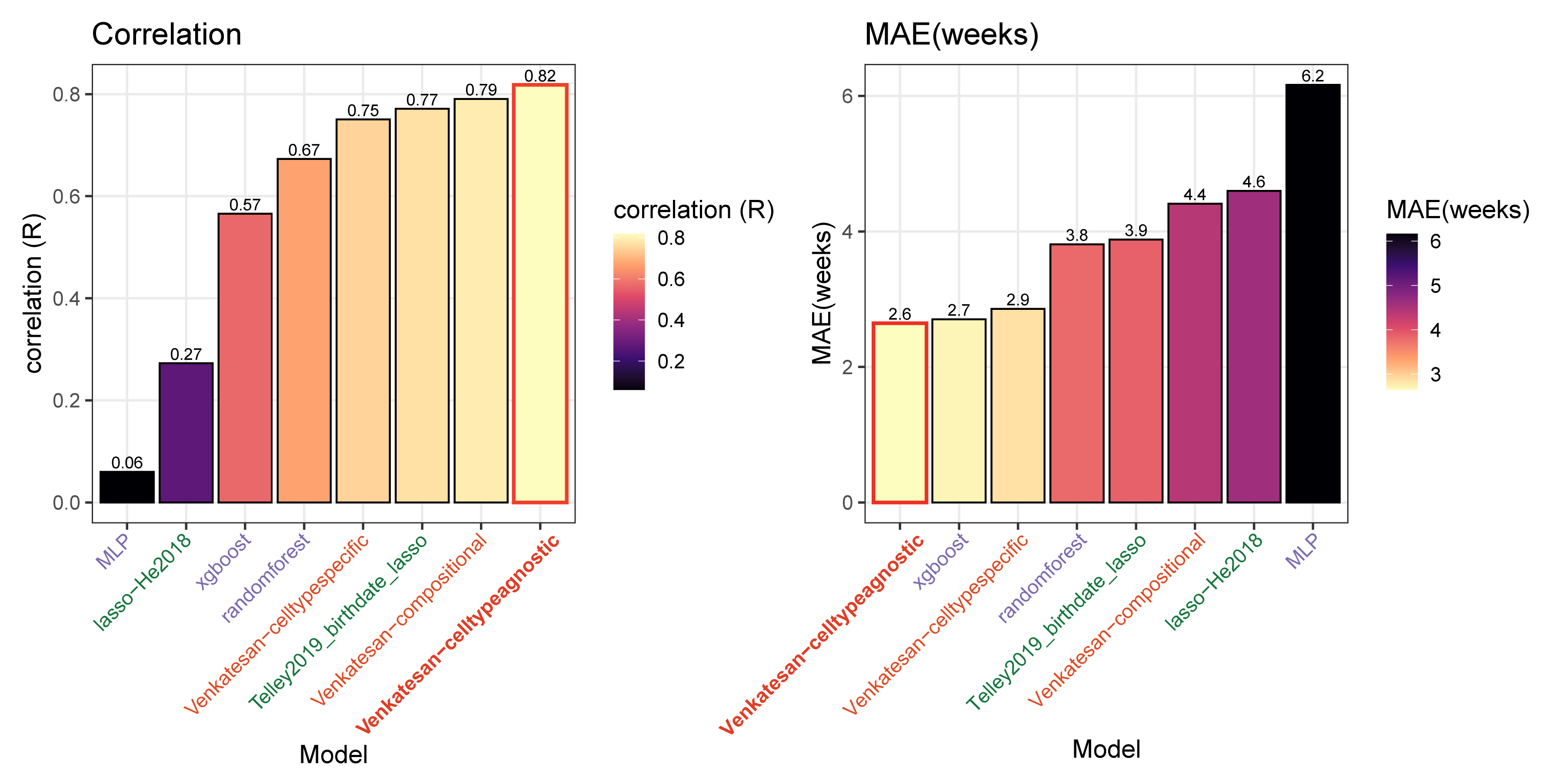

Supplement: S8 Fig — Plots show median correlation and mean absolute error (MAE) in cross-validation for each of the models on the x-axis. Venkatesan-celltypeagnostic, Venkatesan-celltypespecific, and Venkatesan-compositional are models developed in this paper. MLP, xgboost, and randomforest are more complex nonlinear models trained on our data. Lasso-He2018 and Telley2018_birthdate_lasso are models trained on our data but using gene sets derived from previous studies as external comparisons. (TIF) [file pbio.3003757.s008.tif]

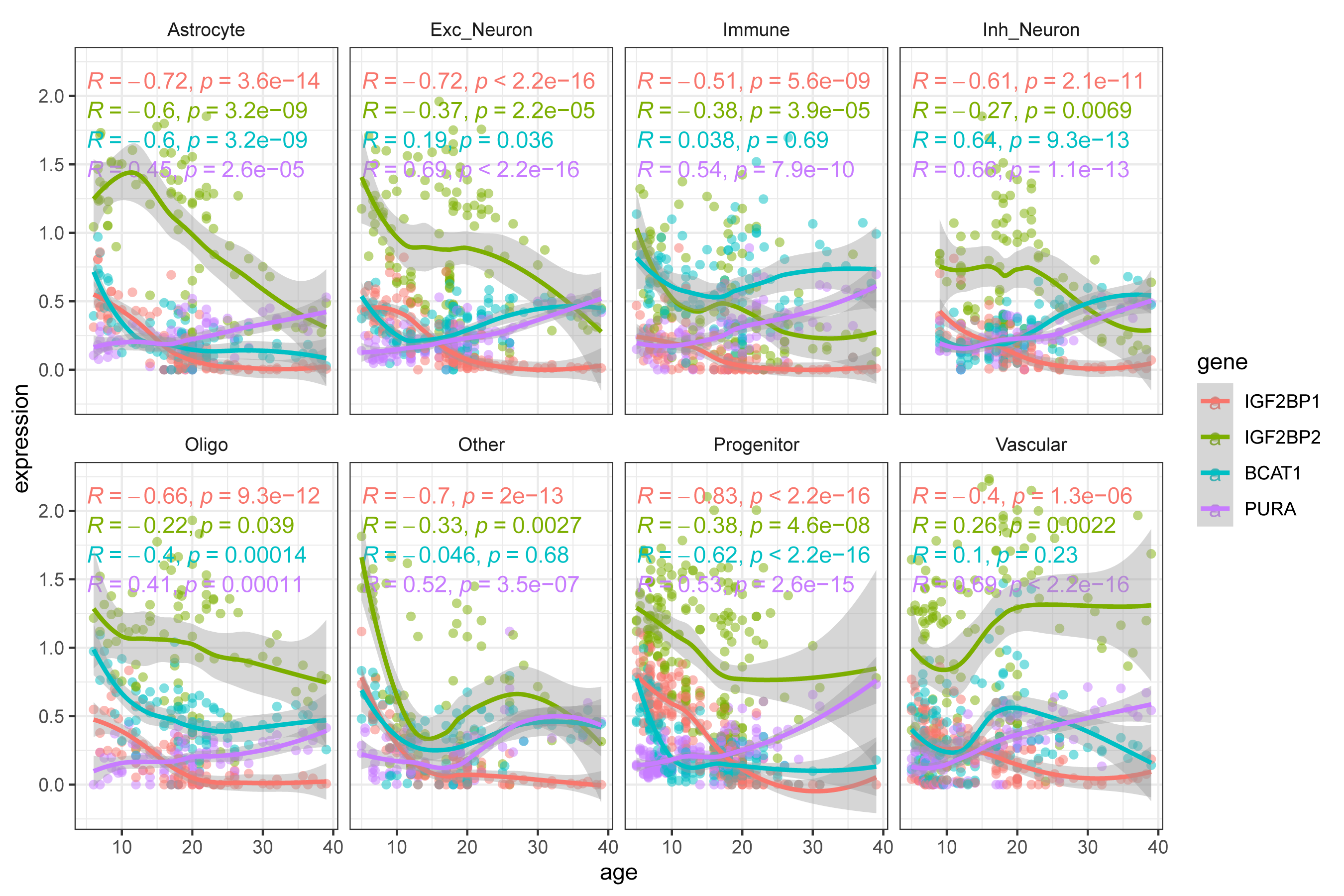

Supplement: S9 Fig — Plots show average log-normalized expression of 4 cell type-agnostic model genes over age in different cell types. Each gene shows distinct and dynamic expression trends across cell types. IGF2BP1/2 are generally negatively correlated to age, PURA is positively correlated, and BCAT1 shows variable expression trends across cell types and developmental windows. (TIF) [file pbio.3003757.s009.tif]

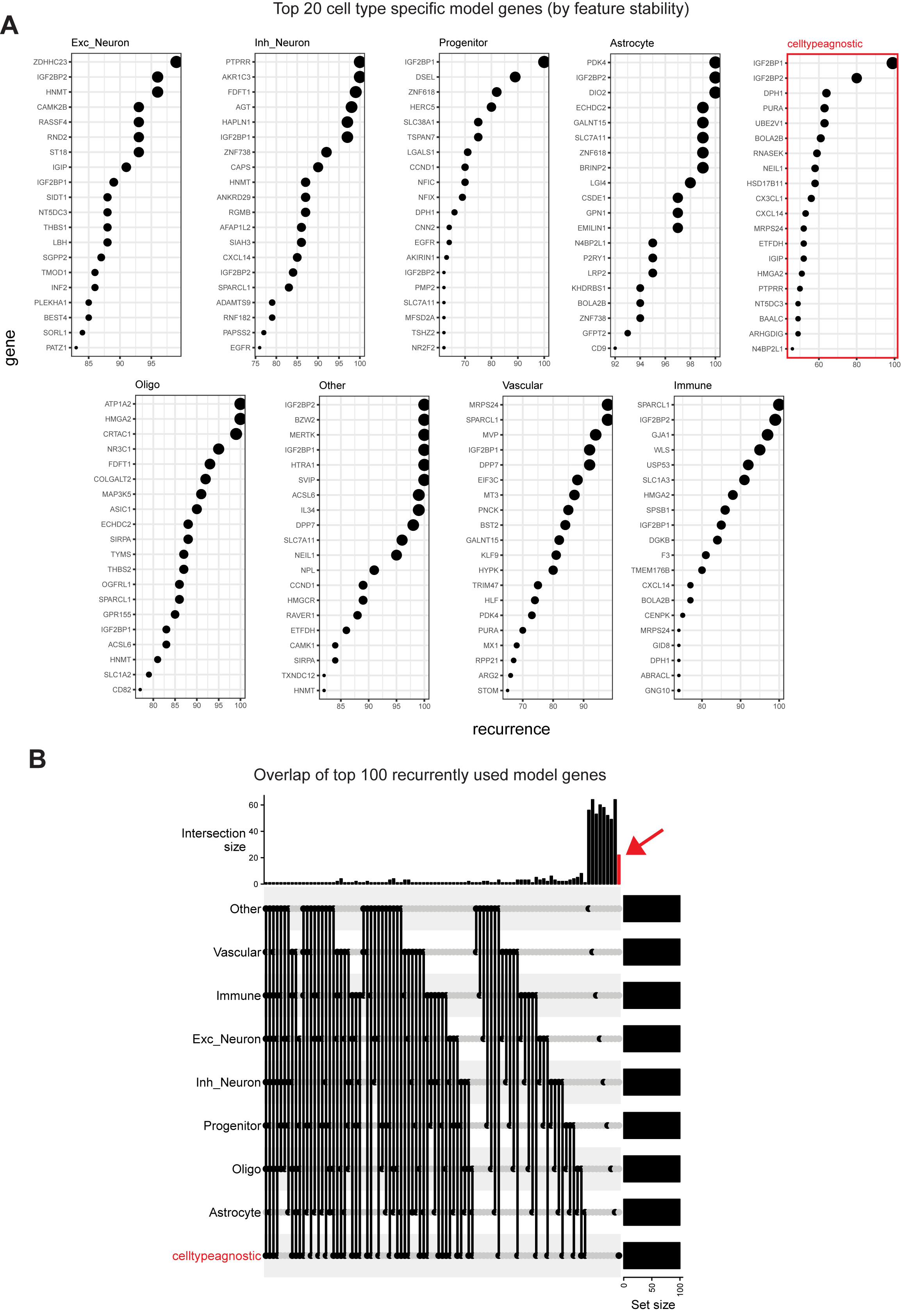

Supplement: S10 Fig — A, Top 20 genes per model identified by feature stability: i.e., recurrent usage across 100 models trained on resampled data. B, UpSet plot showing overlap of top 100 model genes across cell type-specific and cell type-agnostic models. The red arrow points to smaller number of unique genes used by the cell type-agnostic model compared to cell type-specific models. (TIF) [file pbio.3003757.s010.tif]

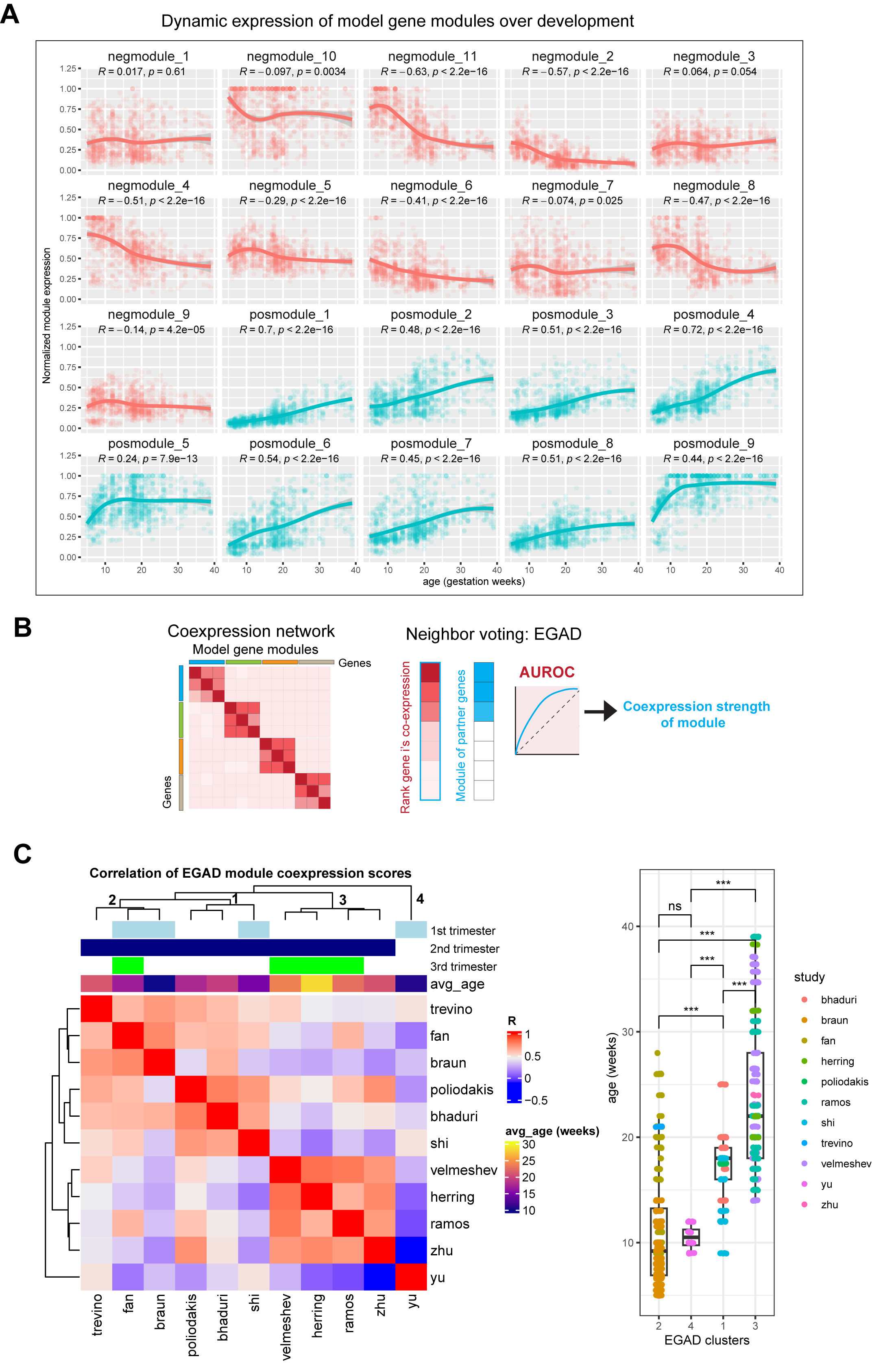

Supplement: S11 Fig — A, Dynamic expression trends of each module from the 462 cell type-agnostic model genes. Plots show normalized average expression of genes per module. B, Schematic of EGAD pipeline to determine co-expression strength of a gene set. C, Correlation of module EGAD scores between studies groups datasets by age. Studies sampling similar temporal windows group together. (TIF) [file pbio.3003757.s011.tif]

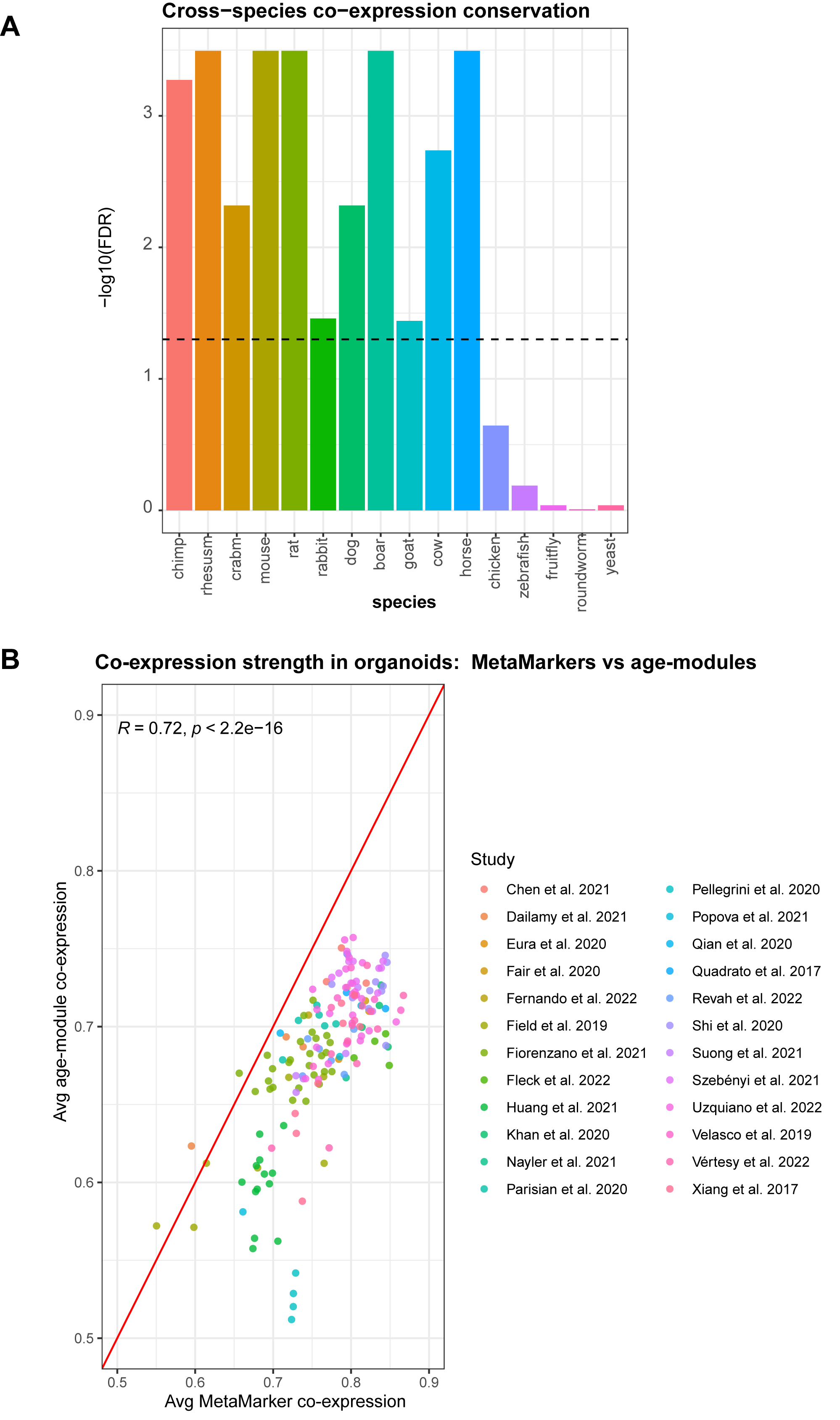

Supplement: S12 Fig — A, Cross-species co-expression conservation of cell type-agnostic model genes measured by −log10 (FDR) compared to random gene sets. Model gene co-expression is highly conserved across mammals. B, Correlation of developmental module co-expression scores from our cell type-agnostic model and fetal cell type MetaMarker co-expression from Werner and Gillis 2,024 in multiple organoid datasets. Developmental modules show generally lower co-expression than cell type modules, but both are tightly correlated across 24 organoid datasets. (TIF) [file pbio.3003757.s012.tif]

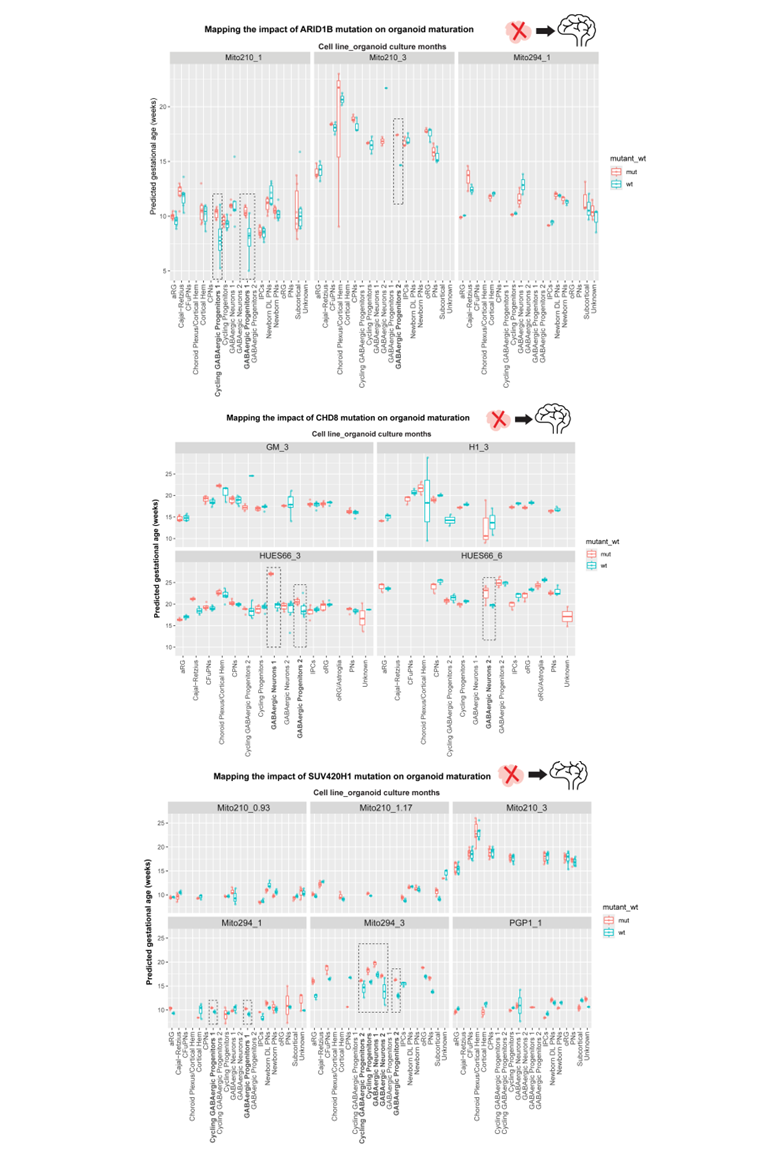

Supplement: S13 Fig — Predicted ages in each cell type from Paulsen and colleagues 2022 neural organoids separated by gene, cell line, and organoid culture age (in months). Dotted boxes highlight GABAergic cell types where mutant cells show accelerated maturation, as per the original study. (TIF) [file pbio.3003757.s013.tif]

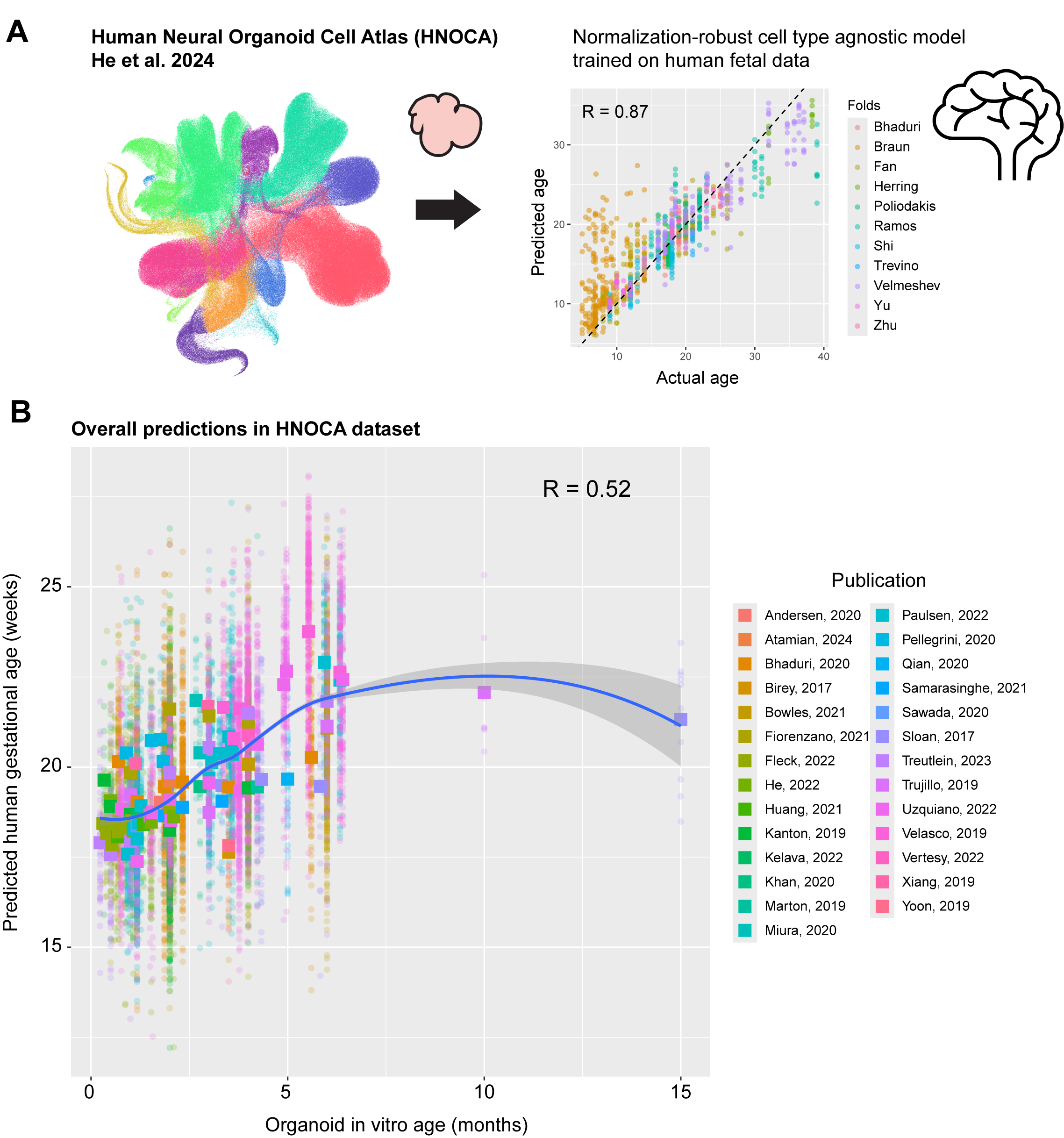

Supplement: S14 Fig — A, UMAP of HNOCA cells taken from CellxGene browser (left). The right panel depicts cross-validation performance of cell type-agnostic model trained on rank-normalized expression values from human fetal brain cell types. B, Developmental age predictions for organoid cells from different studies in the integrated HNOCA dataset. Predicted age is overall strongly correlated to organoid culture age in vitro. Square points show mean predicted age of all cell types per time point in each study. (TIF) [file pbio.3003757.s014.tif]

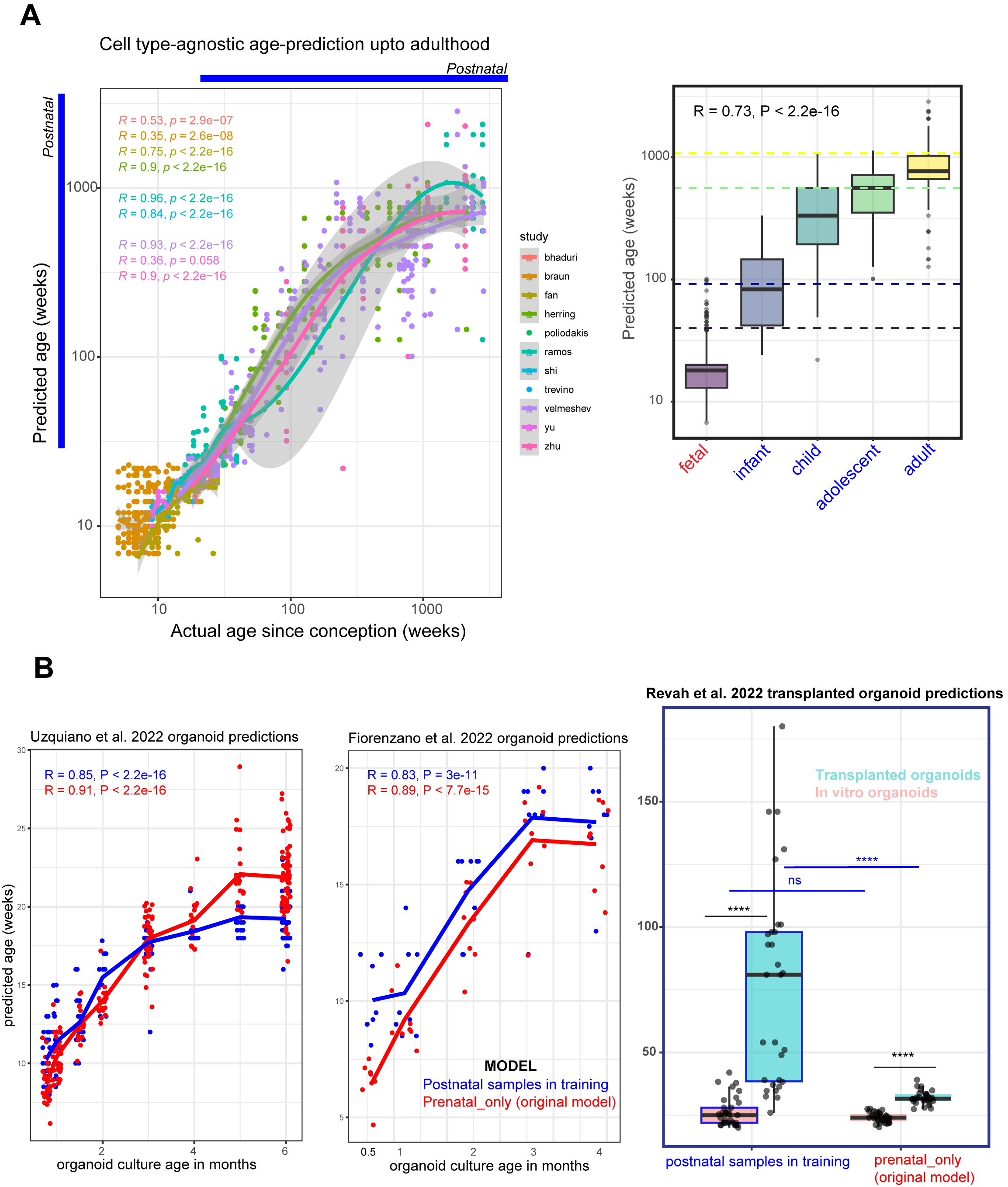

Supplement: S15 Fig — A, Cross-validation performance of cell type-agnostic age prediction model trained on both prenatal and postnatal data from 11 datasets. Predicted ages are strongly correlated with actual age (weeks since conception). Boxplots show predicted ages grouped by developmental windows: prenatal (<40 gestation weeks), infancy (<1year postnatal), childhood (<10 years), adolescence (<20 years), and adulthood (>20 years). B, Developmental age predictions for neural organoids using the original cell type-agnostic model trained on prenatal data only (red) and the updated model trained on prenatal plus postnatal data (blue). In vitro organoids show a plateau in predicted age at ~20 gestational weeks with both models, whereas transplanted organoids exhibit substantially extended maturation with the updated model, reaching predicted ages of up to 1–2 years postnatally (****P < 10−4, ns: not significant, FDR-adjusted Wilcoxon P-value) (TIF) [file pbio.3003757.s015.tif]

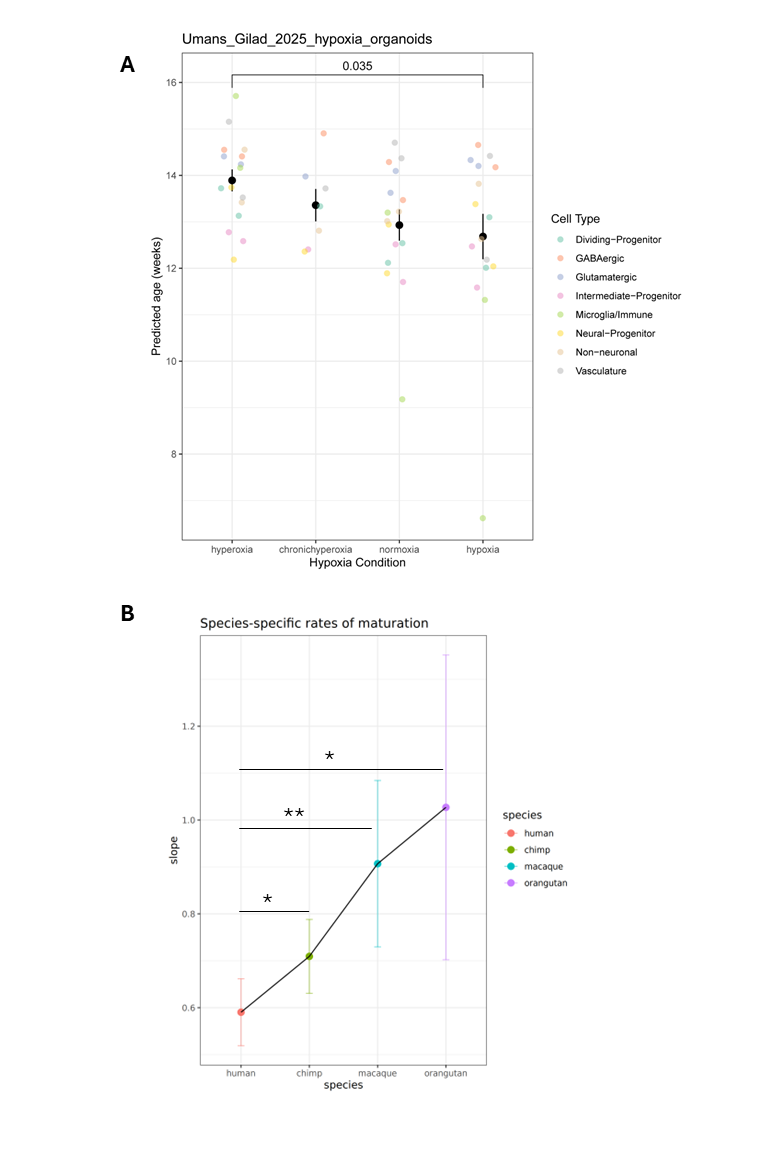

Supplement: S16 Fig — (Top) Cell type-agnostic age predictions show significant delay in maturation in neural organoids grown in hypoxia compared to hyperoxia in dataset from Umans and Gilad 2025. (Bottom) Species-specific rates of maturation measured by the slope between predicted and actual age in interspecies midbrain organoids from Nolbrant and colleagues 2024. Slope and 95% CI are shown as error bars. Human cells show slowest rate of maturation while other primates are faster. *P < 0.05, **P < 0.01, t test comparing slopes from linear model. (TIF) [file pbio.3003757.s016.tif]
