# Supplementary material for: Cell type-agnostic transcriptomic signatures enable uniform comparisons of neural maturation
Source: PLoS Biol. 2026 Apr 15;24(4):e3003757. doi: 10.1371/journal.pbio.3003757 (PMC13095120; doi:10.1371/journal.pbio.3003757)
Supplement: Attachment — Submitted filename: Response to reviewers_final_PLOSBiology.pdf [file pbio.3003757.s026.pdf]

Dear Dr. Hart,

Thank you for the careful evaluation of our manuscript, *“Cell Type-Agnostic Transcriptomic Signatures Enable Uniform Comparisons of Neurodevelopment,”* at *PLOS Biology*. We appreciate the reviewers’ thoughtful feedback and are grateful for their recognition of the value of a generalizable framework for aligning developmental timing across systems. Reviewers noted that “this is an interesting exploration and provided a summary model that is applicable to other studies” [Reviewer 1], that “the predicted ages can serve as a clock to assess developmental timings across different studies, which is of significant interest to the community” [Reviewer 3], and that “these models provide a practical framework for aligning neurodevelopmental tempo across systems,” highlighting “the elegant linear mapping between mouse embryonic days and human gestational weeks” [Reviewer 4].

We also appreciate your suggestion to revise the manuscript for the *Methods* category, which we agree best reflects the primary contribution of the work. The goal of this study is not to advance a single mechanistic pathway, but to introduce and rigorously validate a transparent, meta-analytically trained approach for uniform developmental staging across heterogeneous single-cell datasets, organoids, and species. We have revised the manuscript accordingly, with a stronger emphasis on methodological clarity, robustness, interpretability of the learned temporal signature, and demonstrated utility.

In response to the reviews, we have made several substantive additions. We clarified and corrected our feature-importance analysis and expanded the biological interpretation of the resulting gene set and co-expression dynamics; conducted sensitivity analyses requested by the reviewers, including training-size and dataset-composition controls; benchmarked performance against existing gene sets and alternative model classes; and incorporated additional downstream applications and use cases suggested by the reviewers, including hypoxia and expanded cross-species organoid analyses. We believe these revisions directly address the key concerns raised while reinforcing the manuscript’s contribution as a broadly useful methodological resource. A detailed point-by-point response follows below.

Best,

Jesse Gillis, on behalf of all the authors

REVIEWS:

**Reviewer #1:** In this manuscript, the authors presented their meta-analysis on publicly available primary human developing brain scRNA-seq data to explore age-related features, and to establish age predictors given tissue composition or pseudobulk transcriptomic profiles. The authors also showcased how the obtained cell type-asgnostic age predictor can be applied to human neural organoids and mouse brain data to reveal developmental progression. This is an interesting exploration and provided a summary model that is applicable to other studies. On the other hand, the biological insight presented in the current manuscript are not really outstanding and novel. Some interpretations of the models are questionable and require additional clarification or validations. In addition, the method used to establish the models are not very innovative. The use case examples are valid and provide useful summarized information, but can't provide detailed and mechanistic insight, and thus represent only limited applications.

Considering the mentioned limitations of the current manuscript, I'm afriad that I couldn't be more positive on the manuscipt, at least for its current form. In my opinion, it needs great improvement, either on the biological insight or on the technical innovation, to be able to get accepted in PLOS Biology.

We thank the reviewer for their careful evaluation and for noting that the study provides “an interesting exploration” and “a summary model that is applicable to other studies.” We agree that the primary contribution of this work is not the introduction of a new algorithm or the dissection of a single mechanistic pathway, but the development and validation of a robust, generalizable framework for aligning developmental timing across heterogeneous datasets and experimental systems.

In line with the editor’s recommendation, we have revised the manuscript for the *Methods* category, which we believe is the most appropriate context for this contribution. In the revision, we place greater emphasis on methodological clarity, robustness, and interpretability, and we expand benchmarking against existing gene sets and alternative model classes.

To address concerns about biological interpretability and use, we also added analyses that clarify the biological signal captured by the cell type-agnostic model (including revised feature-importance analyses and co-expression dynamics), and we expanded downstream applications, including reviewer-suggested use cases in hypoxic organoids and additional cross-species systems. These additions are intended to demonstrate how the method can be used to generate biologically meaningful comparisons, while remaining within the scope of a broadly applicable methodological resource.

Followings are my detailed comments.

1. The tissue-level analysis is generally solid. The results are not surprising, that study-specific differences impact generalization. I would expect more about searching for and discussion about the technical or biological factors that affect such generalization. Is it mostly due to regional differences? Or is it mainly the technical variations, such as the dissection procedures that different studies may have dissected different parts of the tissues which have different enrichment of cell types? This is also relevant to the finding by the authors that astrocyte and progenitor proportions can predict well the developmental age, as astrocytes migration during development are reported to be more erratic (Tabata, Nat Comm. 2022). Also, as the analysis was based on the author-provided metadata, the authors should firstly verify whether the cell type annotations by different authors are compariable without needing additional effort to harmonize.

We agree that identifying the factors limiting cross-study generalization of composition-based age prediction is important. In practice, however, batch effects in single-cell and single-nucleus RNA-seq arise from many heterogeneous and partially unobserved sources, including dissociation protocols, nuclei isolation, library preparation, and sample handling, which vary unpredictably across studies and technologies. Because the datasets we analyze span multiple platforms (single-cell, single-nucleus, Drop-seq, STRT-seq), it is not feasible to attribute generalization failure to a single technical or anatomical factor using metadata alone.

To directly test the reviewer's hypothesis that modest compositional differences can disrupt age-associated tissue-level signals, we performed a sensitivity analysis in which we removed one major cell type at a time from each study, recomputed pseudo-bulk expression, and re-derived principal components. We then assessed whether the age-associated PC remained stable. We find that removing a single lineage is sufficient to alter or abolish the age-correlated PC in 4 of 8 datasets (**Supplementary Fig. 5**). This mirrors our cross-study projection results and demonstrates that even moderate differences in cell type composition can substantially alter PC structure, explaining why tissue-level PCs often fail to generalize across studies.

In contrast, astrocyte–progenitor proportions remain predictive of developmental age across all datasets. While astrocyte migration has been reported to be spatially heterogeneous during cortical development (Tabata et al., 2022), our compositional models operate at the level of whole-sample cell type proportions and do not distinguish finer anatomical subregions (e.g., ventricular versus cortical plate). This coarse aggregation likely contributes to their robustness across studies.

We also addressed the reviewer's concern regarding reliance on author-provided cell type annotations. To assess annotation consistency, we evaluated cross-study cell class replicability using MetaNeighbor. Grouping author annotations into seven major classes, we find high replicability across the nine fetal brain datasets used for training (mean AUROC: Astrocyte = 0.95, Neuron = 0.80, Immune = 0.86, Oligodendrocyte = 0.90, Progenitor = 0.84, Vascular = 0.87), with lower scores only for the heterogeneous "Other" category (0.63). In a more stringent one-vs–next-best comparison, replicability remains high (mean AUROC = 0.68). These results (**Supplementary Fig. 2**) indicate that cell class annotations are sufficiently consistent at the resolution used for tissue-composition modeling.

Supplementary figures 5 and 2 are shown below, the response to reviewers comments continues on Page 6.

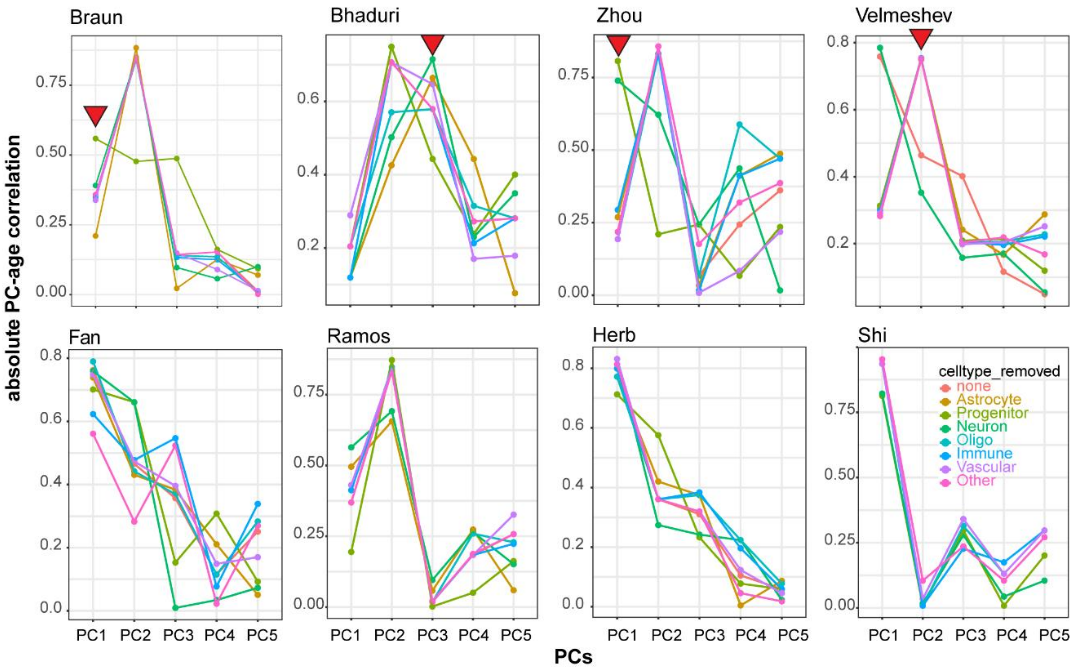

**Supplementary Figure 5 Sensitivity of Principal component age predictions to cell type removal.** Panels show the absolute correlation coefficient between principal components (PC1-PC5) and age in each dataset. PCs were computed from pseudo-bulked gene expression. Colors indicate PCs recomputed after removing all cells of a specific cell type from the dataset. Removing cell types shifts the PC-age correlation in 4/8 datasets shown in the top row. Red arrows indicate complete change in the PC-age correlation: i.e. in Braun et al., removing Progenitors from the dataset shifts the age-correlation from PC2 to PC1. In Zhou et al., removing Progenitors or Neurons from the dataset shifts age-correlation from PC2 to PC1.

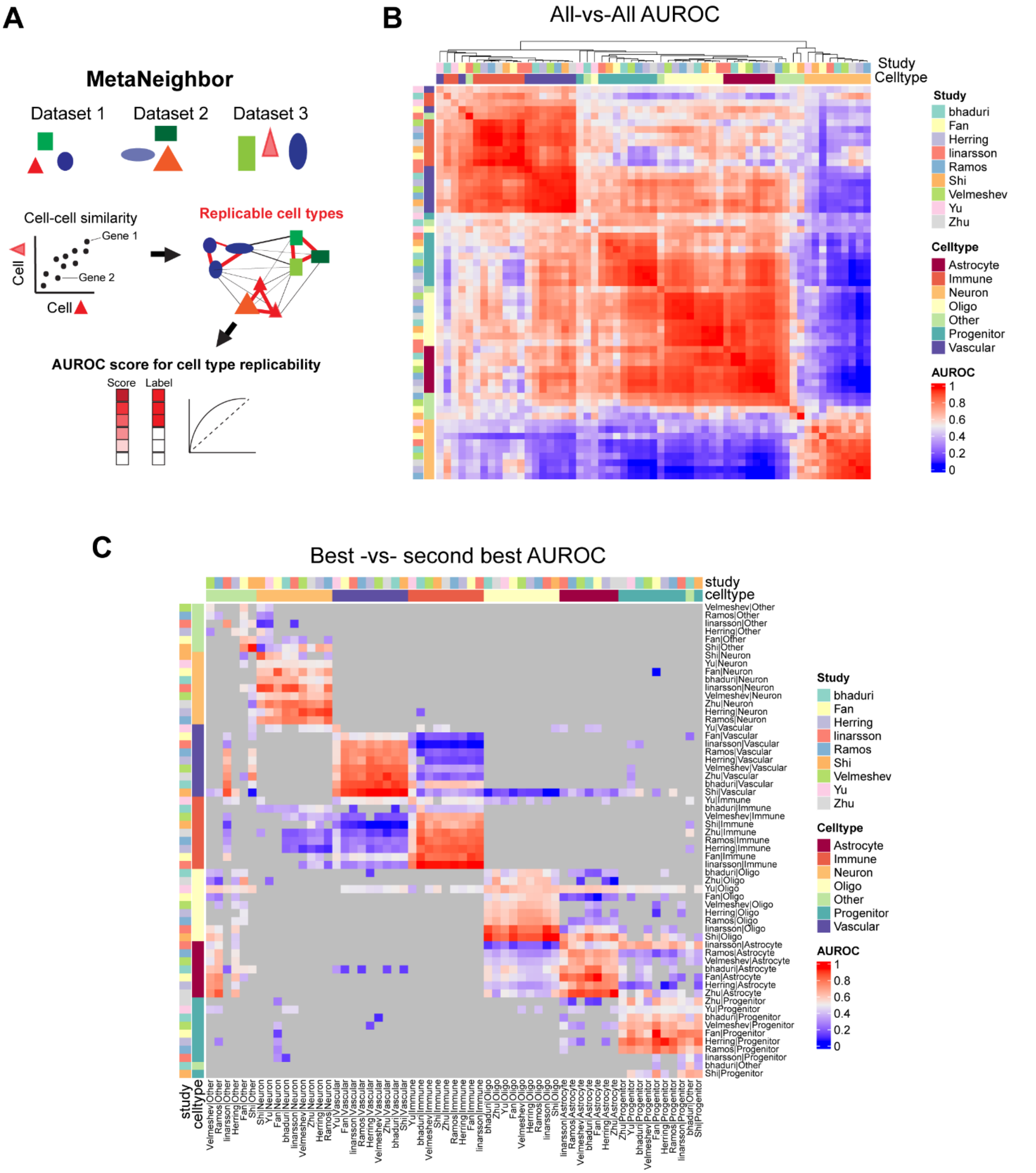


**Supplementary Figure 2: MetaNeighbor assessment of cell type replicability in fetal brain single-cell datasets. A,** Schematic illustrating metaneighbor procedure: cell similarity networks constructed from gene-gene correlation are used to assess cell type replicability with a leave-study out cross-validation approach. The AUROC score measures whether cells of the same type rank higher than other cell types in the network. **B,** Heatmap shows MetaNeighbor AUROC scores for all vs all comparisons of major cell classes across 9 datasets used for model training. Cells cluster by cell type rather than study **C,** Heatmap shows best vs next best MetaNeighbor

AUROC scores measuring the ability to discriminate cells of the same type from the next closest cell type. Cells cluster by cell type rather than study.

2. The regularized regression model used to establish the age prediction models are powerful and commonly used, but it is an old school method without substantial technical innovation, and it has been used before for relevant topics, e.g. neuronal maturation (He, BMC Genomics 2018). Of course, the algorithm behind the scenes doesn't need to be new and fancy, but without substantial technical innovation, in-depth biological insights should be presented. Although the authors did try to look into the established model and interpert its biological implication, the reported results are not very exciting either, and no biological followup was done or proposed.

We agree that regularized regression is a well-established and powerful approach for feature selection. The novelty of our work does not lie in the algorithm itself, but in how it is used: we integrate 2.8 million cells across 13 studies, technologies, and brain regions to train a single meta-analytic model of developmental time. This large and heterogeneous training corpus provides a level of robustness and generalizability that prior single-study models could not achieve.

To directly address the reviewer's concern, we benchmarked our model against prior developmental or maturation gene sets:

**(1) Comparison to He & Yu 2018 neuronal maturation modules.**
He & Yu [1] reported 33 gene modules (2136 genes) derived from a single cortical dataset of 466 cells [2]. Because pretrained models are not available, we retrained LASSO models using their module genes on our meta-analytic data. All module-based models performed significantly worse than our cell-type-agnostic model (FDR-adjusted Wilcoxon $P < 0.05$). Mean prediction errors ranged from 4.08–5.65 weeks versus 2.4 weeks for our model. The modules also showed far greater inter-study variability (variance in MAE = 5.83 weeks vs. 0.81 for our model). These results illustrate that models trained on limited single-study data do not generalize well, even when retrained on our much larger corpus.

**(2) Comparison to the Telley et al. 2019 birthdate model.**
We compared our model to birthdate models from Telley et al. 2019 [3], which were trained on embryonic mouse brain data (E12-E15) with 2756 cells using regularized ordinal regression. The published ordinal regression model did not generalize to our human datasets, yielding significantly weaker correlations between predicted and actual age (Wilcoxon $P = 0.00185$). A retrained model using the Telley gene set and LASSO also underperformed (mean error 4.06 vs. 2.4 weeks, FDR-adjusted $P = 0.005$). This further supports that gene sets derived from narrower developmental windows or species do not capture broad, cross-context developmental progression. These benchmarking results are shown in **Supplementary Fig. 7**.

Although our method is simple, we go beyond prediction accuracy to interpret the biological signal. We identify top genes contributing to model performance as global regulators of mRNA dynamics (*IGF2BP1*/*2*), elaborated further in point 4. We show that our model genes display coherent temporal co-expression dynamics across fetal development and form conserved co-expression modules across mammalian species (**Supplementary Fig. 11-12**). These results indicate that our model leverages highly conserved cellular processes to track developmental time across contexts. Furthermore, the model yields biological insight in novel contexts: it

accurately detects delayed maturation in hypoxic organoids [4] and recapitulates species differences in developmental tempo from interspecies midbrain organoids [5]. These applications are included as **Supplementary Fig. 16** and further explained in response to Reviewer #4.

This work represents a parallel and complementary advance to recent deep-learning approaches such as TemporalVAE [6], offering a transparent and easily deployable alternative trained on an unprecedented meta-analytic corpus. In accordance with the editor's suggestion, we are resubmitting our revised manuscript as a Methods and Resources article.

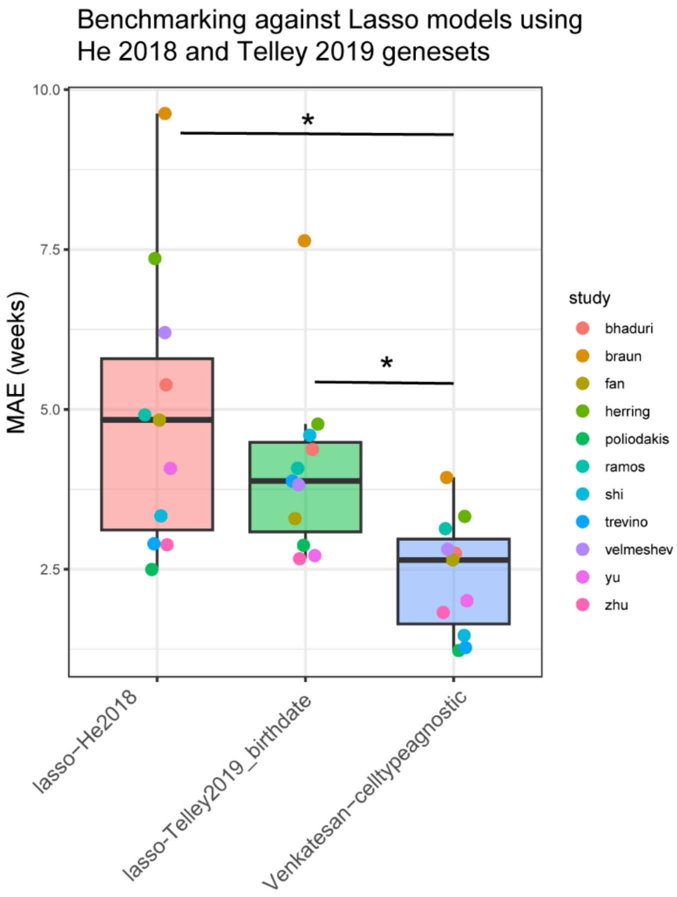


**Supplementary Figure 7. Benchmarking performance of cell type agnostic model against maturation modules from other studies.**

L1 (LASSO) regularized regression models to predict age were trained using neuronal maturation gene sets from He et al.2018 and Telley et al.2019. Boxplots compare prediction accuracy (mean absolute error, MAE) in each of the human fetal datasets to the cell type-agnostic model developed in this study. (* adjusted Wilcoxon P < 0.05)

3. The finding that the cell type-agnostic model performs better than the cell-type specific models is an interesting finding. The authors proposed that it was because the cell type-agnostic model, by including data of different cell types, captured different age-related genetic programs. However, another technical explanation is that the cell type-agnostic model was trained using way more data than the cell type-specific models. The authors should firstly show that the cell type-agnostic model performs better with the training dataset size controlled.

We thank the reviewer for this great suggestion and agree that this is an important control experiment. We retrained 100 models on randomly sampled subsets of 30, 50 or 70 samples from the original training data for both cell type-specific and cell type-agnostic models to control for training dataset size. As expected, increasing training-cell number consistently improved

model performance across all models (e.g. mean error = 3.9, 3.2 and 2.7 weeks across all cell types for the cell type-agnostic model with N= 30,50,70)

When training size was held constant, the cell type-agnostic model did not necessarily achieve the lowest error within each individual cell type: for example, the astrocyte-only model performs better on astrocytes (mean error = 0.58 vs. 2.84 weeks for the cell type-agnostic model). But the cell type-agnostic model is still the second-best performing model for all cell types, regardless of number of cells used in training. The main point is that the cell type agnostic model maintains substantially stable performance and lower error across all cell types than single cell-type specific models.

This pattern is also evident in the hypothalamus test datasets (**Supplementary Fig. 6A**), where the agnostic model shows similar prediction error across all cell types, whereas cell-type-specific models perform well only in the cell type they were trained on and poorly on others. Consistent with this, one-way ANOVA across test cell types shows that the cell-type-agnostic model has the lowest between-cell-type variance (F = 9.63), whereas all other models exhibit substantially higher F-values (F = 26–146), demonstrating that the agnostic model generalizes far more uniformly across diverse cell types. This stability is particularly important for the use-cases we present such as prediction in novel organoid model systems or cross-species applications, where matching or defining corresponding cell types can be challenging.

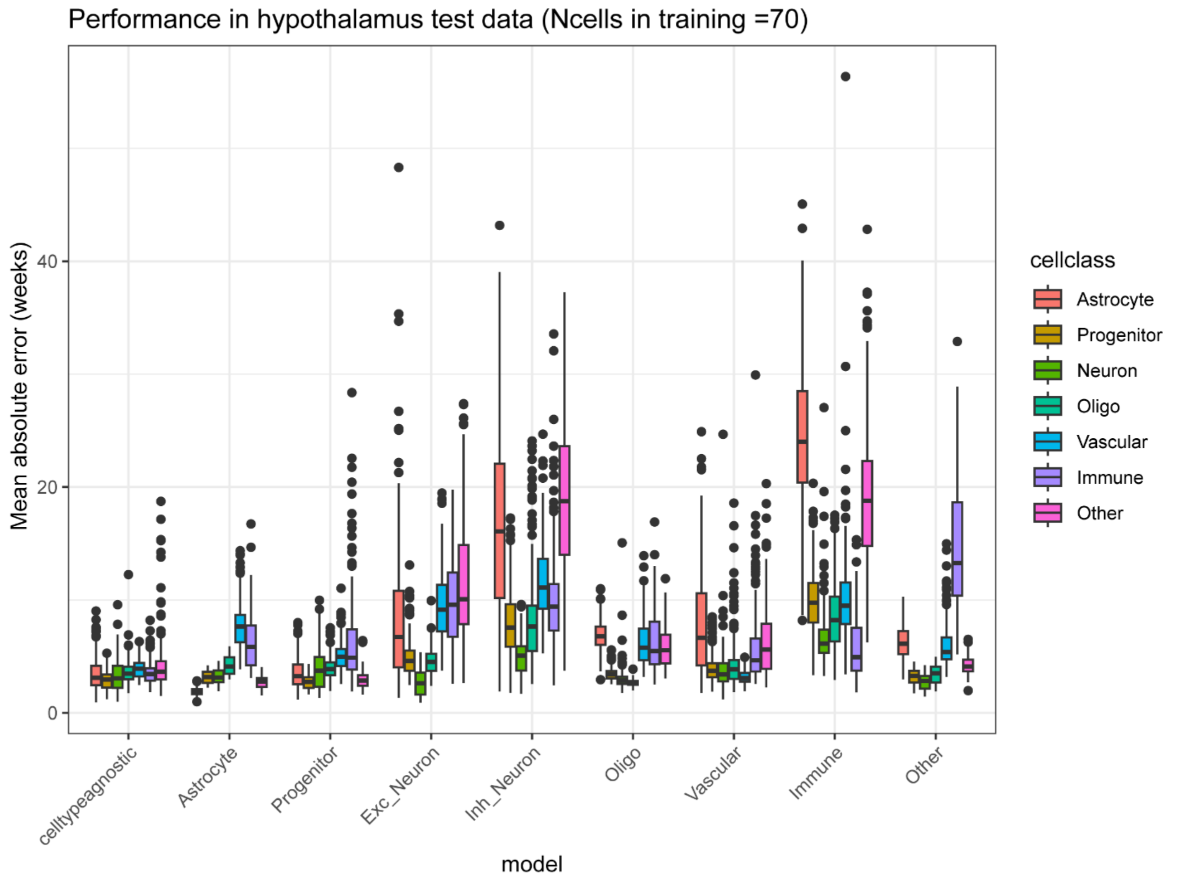


**Supplementary Figure 6A. Distribution of prediction errors for cell type-specific and cell type-agnostic models controlled for training data size.** Boxplots show distribution of

prediction error (Mean Absolute Error, MAE) for 100 cell type-specific and cell type-agnostic models were trained on the same number of cells in training (N = 70).

4. The genes selected by the cell type-agnostic model are confusing. Many of genes reported in Fig.4A with the highest importance, such as OAZ3, VMO1, AMIGO2 and CEL, don't show high expression in any of the developing brain cell types in both mouse (La Manno, Nature 2021) and human (Braun, Science 2023). This makes me wonder how much biological insight one can actually get by looking into those genes, and how reliable they are.

We thank the reviewer for pointing this out. In the original manuscript, we mistakenly reported the raw model coefficients as feature importance. Because coefficients are scaled inversely with gene expression, this unintentionally highlighted lowly expressed genes (e.g., OAZ3, VMO1), leading to the confusion raised by the reviewer. We have now replaced this approach with two established measures of variable importance: permutation importance and feature stability [7,8]. Permutation scores are computed for each gene by permuting its expression across all cells and then evaluating the drop in age prediction performance, while feature stability measures how often a gene is used across models trained with repeated subsampling of the training data. These metrics are correlated for the cell type-agnostic model ($R = 0.41$, $P < 2.2\times10^{-16}$) and produce biologically interpretable top features.

Using these revised metrics, the highest-importance genes are enriched for **m6A RNA reader activity and CRD-mediated mRNA stabilization**, driven by ***IGF2BP1*** **and** ***IGF2BP2*** (**revised Fig. 4A**)**.** IGF2BPs (also known as Imp) are conserved RNA binding proteins that act as readers of m6A mRNA methylation and regulate mRNA stability, translation, and localization; their strict temporal expression gradient controls the timing of neurogenesis and neuronal fate transitions across species [9–16], and their disruption impairs cortical layer formation [17,18]. Paralleling its developmental downregulation (**Supplementary Fig. 9**), *IGF2BP1* shows the largest increase in DNA methylation across human fetal cortical development [19], supporting its role as a temporal regulator of cell fate.

Apart from IGF2BPs, top genes used by the cell type-agnostic model include regulators of neural stem cell proliferation (***BCAT1, PURA***) [20–22]; mitochondrial metabolism (***ETFDH***) [23] and amyloid beta processing (***CLU***) [24]; Transcription factors with known roles in neural fate specification (***KLF9, SOX15, POU3F2***) and neuronal signaling genes such as ***CAMK2B*** and ***KCNN2*** also rank highly [25–28]. Overall, our revised metrics reveal a coherent set of genes with established roles in neurodevelopment and cell fate. These details have been added to the main manuscript.

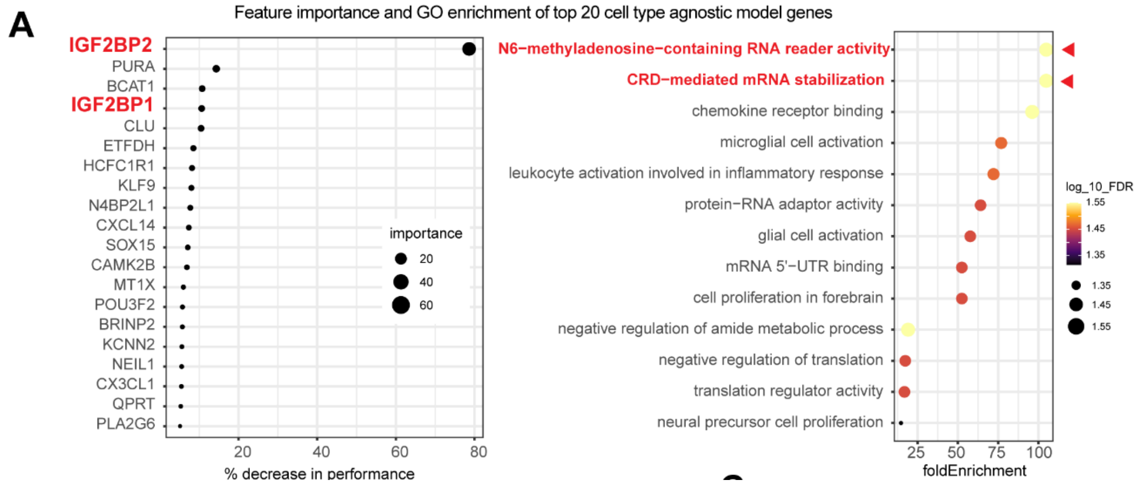


**Revised Figure 4A. Top 20 features of cell type agnostic model. (Left)**Top model genes ranked by their perturbation impact. **(Right)** GO enrichment for top 20 genes highlights m6A mRNA reader activity as an enriched term, mediated by *IGF2BP2* and *IGF2BP1*.

5. The authors looked into genes selected by the cell type-agnostic model for the enriched biological functions, which I appreciate. Meanwhile I think more should be done by looking into differennces and similarities of those biological processes among different cell types, and also to compare with the genes selected by different cell type-specific models. In my opinion this is very crucial, considering that the enriched functions are predominately neuron related, while it shows good performance also in the non-neuronal cell types. In fact, this concern, together with my last two comments, make me wonder whether there is a possibility that the cell type-agnostic model actually captures the ambient RNA background which is constance across cell types, likely different from one sample to another with the average transcriptomic changes across ages embedded, and probably neuron-biased (as neurons are more fragile and more likely to be damaged during tissue dissociation).

We appreciate the reviewer's request for a deeper comparison of genes used by the cell type-specific models and the cell type-agnostic model. Using feature stability as described above, we computed variable importance for each cell-type-specific model and present the top 20 genes per model in **Supplementary Fig. 10.** Across models, ***IGF2BP1*** and ***IGF2BP2*** consistently emerge among the top features, indicating that shared RNA regulatory programs contribute to temporal progression across all lineages. In addition, each cell-type-specific model also identifies well-established cell-type-specific markers, such as transcription factors *NFIX* and *NFIC* in progenitor cells [29,30]; *CAMK2B* in excitatory neurons; synaptic vesicle gene *CAPS* and perineuronal net component *HAPLN1* in inhibitory neurons [31–33]; the Cystine-glutamate transporter *SLC7A11* in astrocytes [34,35]; Regulators of glial cell development (*NR3C1* and *COLGALT2*) in Oligodendrocytes [36,37]; *MVP*, an endothelial protein and stomatin (*STOM*) a marker of red blood cells in vascular cells [38,39] ; Connexin 43 (*GJA1*) and Wnt ligand secretor (*WLS*) in Immune cells [40]. GO enrichment of the top features per model confirms this diversity, identifying progenitor-specific cell cycle terms, glial cell migration (astrocytes), regulation of reactive oxygen species & PI3K signaling (inhibitory neurons), glutamate transport (oligodendrocytes), and mesoderm morphogenesis (microglia) among others.

Consistent with a shared temporal signal identified by the cell type agnostic model, examining the overlap of the top 100 genes across models shows that the cell-type-agnostic model has the fewest model-specific genes (n = 22, versus 49-64 for cell type-specific models) and the greatest overlap with genes selected by multiple cell type-specific models. This further supports the fact that our model captures broadly consistent temporal regulators rather than ambient RNA or sample-specific noise. These results are shown in **Supplementary Fig. 10.**

Importantly, the expression patterns of the top features used by the cell-type-agnostic model (**Supplementary Fig. 9**) show robust and cell-type appropriate temporal expression, rather than a uniform low-level signal expected from ambient RNA contamination. Moreover, ambient RNA effects would be expected to introduce sample-specific noise, yet our model generalizes across 13 studies, multiple technologies, and species, conditions under which ambient backgrounds differ markedly. The model's ability to detect delayed maturation in hypoxic organoids and species-specific tempo differences further argues against ambient RNA as the dominant signal.

Together, these analyses demonstrate that the agnostic model captures shared, developmentally regulated RNA programs, while each cell-type-specific model captures additional lineage-specific processes. The enriched functions are therefore not exclusively neuronal; instead, they reflect global temporal regulators combined with cell type-specific temporal signatures, rather than artifacts of ambient RNA.

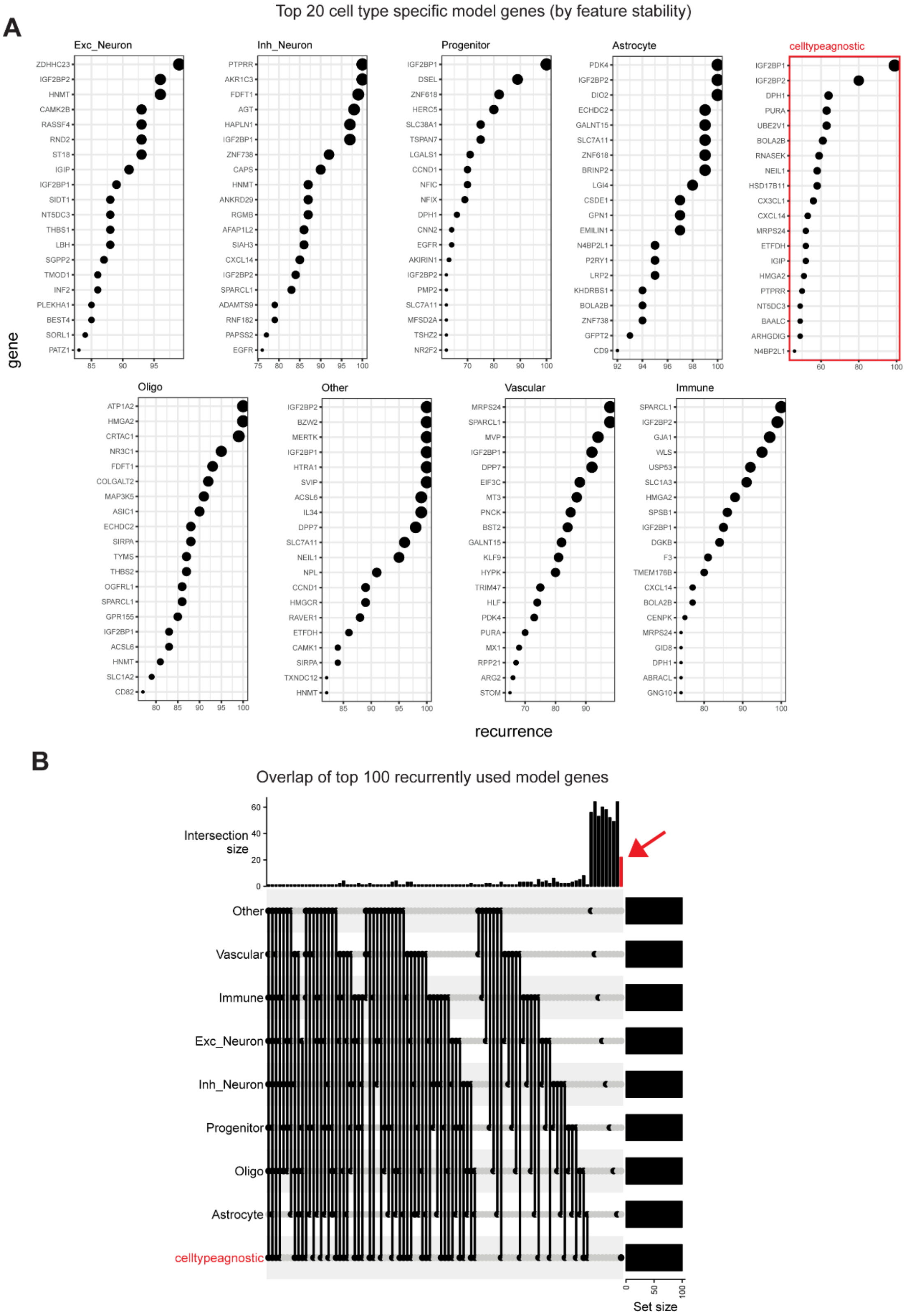


**Supplementary Figure 10: Top genes used by cell type-specific and cell type agnostic models and their overlap. A,** Top 20 genes per model identified by feature stability: i.e. recurrent usage across 100 models trained on resampled data. **B,** UpSet plot showing overlap

of top 100 model genes across cell type-specific and cell type-agnostic models. Red arrow points to smaller number of unique genes used by the cell type-agnostic model compared to cell type-specific models.

6. The authors showed user cases of applying the cell type-agnostic model to human neural organoids and mouse data, which provided reasonable results. My concern here, though, is whether the model actually provides more insight than simplier metrics, such as summarized module scores of maturation related pathways and gene modules (e.g. neuron projection, neurontransmitter secretion). Also, while the predicted ages may act as a useful summary metric, it doesn't seem to help on understanding any mechanism or providing in-depth biological insight.

We agree that simpler maturation metrics, such as pathway or gene-module scores, would be preferable if they were reliable across datasets. To directly address this concern, we explicitly benchmarked our approach against commonly used maturation proxies in neural organoids.

We find that published maturation gene sets perform inconsistently across organoid protocols. Modules from He (2018) and Telley (2019) show only weak correspondence to organoid age (R = 0.23 and 0.27, respectively). Likewise, SynGO-based synaptic module scores do not show consistent positive correlations with culture age, even within projection neuron populations. Across 245 SynGO terms tested, only a single term reaches R > 0.5 in one cortical organoid dataset, and that same term shows a negative correlation with age in midbrain organoids. These results indicate that pathway- or module-based maturation scores do not provide a stable or transferable maturation axis across organoid systems.

Our goal is therefore not to replace mechanistic analyses, but to provide a robust, transcriptome-based developmental staging coordinate that generalizes across protocols, regions, species, and cell types. Because the cell type-agnostic model is driven by conserved temporal regulators and coherent co-expression dynamics, the resulting age estimates reflect shared biological programs rather than arbitrary summary scores, while remaining interpretable and broadly applicable.

**Reviewer #2: Summary**
**The authors aimed to develop a general purpose metric to describe neurodevelopmental stages or developmental age across diverse single-cell datasets. They analyzed 13 single-cell RNA-seq datasets from the developing human brain, across 151 donors and 2.8M cells. Their analysis compares the relative contirbutions of tissue cell type composition and transcriptomic maturation in predicting developmental age.**

**They show that developmental changes in cell type composition can be predictive of developmental age within individual datasets but not across datasets. Since principal components derived from gene expression are also primarily a function of cell type composition, they tested PCs and found that they were not informative for this task. Next, the authors focused instead on specific cell type populations and found that astrocytes and progenitor cells consistently predicted neurodevelopmental age across dataset. In addition, they train a cell type agnostic model to learn a general signature of neurodevelopment. The cell type agnostic model performed better than models trained on individual cell types when evaluating across datasets. It weighed 462 genes as**

**robustly able to predict developmental age, many of which were not strongly correlated with age individually. Lastly, the authors applied their cell type agnostic models to predict developmental progression in human neural organoids and embryonic mouse brain development.**

Comments

1. This paper makes important claims about the capabilities of different models, but it lacks a clear description of what level of performance is meaningful for downstream tasks and evaluation. How much error is acceptable? For instance, what is the difference between 1 week and 6 weeks? In lines 94-95, the authors report a median error of 1 week as an indicator of good performance, while in lines 125-126, a median error of 4 weeks is presented as good performance when training on astrocyte proportion. While I understand that this is relative to performance across cell types, it remains unclear what threshold of accuracy would make the results useful for biological applications.

We agree with the reviewer that model performance must be interpreted relative to the biological question and temporal resolution required. In lines 94-95, the error of 1 week was reported for models trained and tested on the same dataset using leave-sample-out validation; This is expected to achieve high performance, but the model does not generalize, as its error substantially increases (~9 weeks) when evaluated on a held-out study.

To directly relate training accuracy to downstream utility, we evaluated both transcriptome and composition-based models on the neural organoid dataset from Uzquiano et al., using AUROCs to quantify how well predicted ages discriminate organoids at consecutive time points. The transcriptome-based cell-type-agnostic model (median error = 2.8 weeks) correctly discriminated nearly all adjacent organoid ages (AUROC = 1 for all but one comparisons), whereas the composition-based model (median error = 4.5 weeks) showed consistently weaker separation, including AUROCs as low as 0.25–0.38 for early developmental stages. Thus, even modest differences in training error meaningfully affect interpretability in downstream biological applications.

Transcriptome-based cell autonomous models are suitable for fine-grained staging and detecting subtle maturational differences between experimental conditions. In contrast, composition-based models capture slower shifts and provide coarser developmental resolution (~4 weeks). We note that the fetal ages provided by the original studies have an inherent uncertainty of 1-2 weeks (based on estimated conception/ last menstrual period) which constrains the achievable accuracy of any supervised model.

2. The significant difference between the cell type agnostic and cell type specific models seems to indicate that there is much more benefit that these models can obtain from training over larger datasets and being less biased in feature selection. However, training and evaluating is only performed on subsets of 13 datasets and thus does not seem fully conclusive of the claims being made and also whether performance could be significantly improved further. Without such a benchmark, it is difficult to assess whether the reported performance is truly robust across a wide range of datasets.

We appreciate the reviewer's suggestion to benchmark model performance as a function of training dataset size and number of studies included. We performed two complementary analyses to address this.

First, we controlled for training data size by retraining models on subsets of the data with a fixed number of cells. The cell type-agnostic model remains the second-best performing model for all cell types, regardless of number of cells used in training (**Supplementary Fig. 6A**). More importantly, it showed **stable and comparable performance across all cell types**, even when trained only on one-tenth of the full dataset. In contrast, cell-type-specific models performed well in their own lineage and poorly on other cell types. This demonstrates that the advantage of the cell type-agnostic model is not simply due to larger sample size but reflects its ability to capture shared temporal programs across diverse cell types.

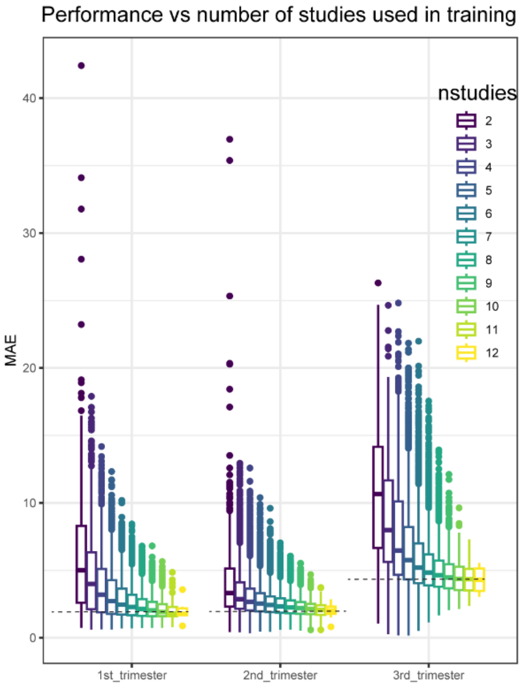


Second, to examine the impact of having heterogenous studies in training, we retrained models on all combinations of studies, using subsets ranging from 2 to 13 datasets and evaluated performance on the remaining held-out studies. Performance improves as more studies are included in training, but the gains plateau after about 6-7 studies, indicating that the model reaches a stable regime of generalization well before all 13 datasets are used. The scarcity of third-trimester cells in all datasets limits accuracy in that window regardless of the number of studies used in training. But models trained on comparable numbers of cells from all trimesters show similar performance in all 3 trimesters.

These results together shown in **Supplementary Fig. 6** demonstrate that the cell-type-agnostic model is robust across a wide range of training conditions and derives its strength from study diversity rather than dataset size alone. Given the scarcity of human fetal brain tissue, our meta-analysis represents one of the most comprehensive evaluations possible at present, and additional studies are not likely to substantially alter the results.

3. Why was the cell type-agnostic model not trained on the full set of protein-coding genes? Did the authors test more complex architectures (e.g. non-linear models) for handling a feature space of over 10,000 genes? Did they try deep learning methods that do not require any feature selection? Furthermore, how did they address cross-species transfer when shared genes were absent?

We thank the reviewer for these questions. Our goal was to develop a model that would generalize across diverse human fetal and neural organoid datasets, so the gene set was restricted to genes (n = 10,957) present in all the datasets in our collection.

In response to the reviewer's suggestion, we trained non-linear and more complex ensemble models, including random forest, XGBboost, and a multi-layer perceptron (10 hidden units). Across all comparisons, our regularized cell type-agnostic model achieved greater accuracy than other complex methods (paired Wilcoxon tests comparing MAE; all FDR adjusted $P < 0.01$, **Supplementary Fig. 8**). These alternative models also required substantially more computation (~0.5 minutes for regularized regression versus 40 minutes for XGBoost, 57 minutes for the MLP, and ~6 hours for random forest) while using more features, limiting interpretability.

For cross-species transfer, we restricted the gene set to one-to-one orthologs shared between human and the target species, as well as genes present in the target dataset and retrained the model. Since the model training takes < 1 minute, retraining on species-specific gene subsets is straightforward and avoids issues with missing genes in new datasets.

4. Given the different models tested, which core model do the authors recommend using? It would be helpful to directly compare the cell type-agnostic model with the models trained on tissue composition. Some evaluations use MAE (in weeks), while others use Pearson R. Consistency in evaluation metrics would strengthen the paper. Could the authors create a final plot comparing all approaches tested for predicting neurodevelopmental age? Additionally, are there existing baseline models for predicting age that could be included for comparison? If the cell type-agnostic model performs best, what advantage does the predictor based on cell type composition provide? Is there a clear benefit of using one over the other?

Given its high accuracy, robustness across datasets, and flexibility in applications where precise cell type matching is not required, we recommend the cell type-agnostic transcriptome model as the core model for downstream analysis.

We now include a summary comparison (**Supplementary Fig. 8**) showing consistent performance metrics (median MAE and Spearman correlation) for all models evaluated in this study. These include our three regression models (cell type-agnostic, cell type-specific, and compositional), three non-linear models (XGBoost, random forest, and MLP), and two baseline models trained on previously published gene sets (Telley 2019; He 2018). **Across all approaches, the cell type-agnostic model achieves the lowest error and highest correlation between predicted and actual age.**

The compositional model provides a coarse, tissue-level indicator of maturation that is interpretable in terms of glial–progenitor balance, but it lacks the precision required for applications involving cell-level developmental shifts. Only the transcriptomic models can reliably resolve subtle differences in neural organoid maturation.

Finally, we note that the cell type-agnostic model has been validated across a broad range of downstream contexts including organoid hypoxia, transplantation, disease mutations, and species differences in chimeric organoids, demonstrating that it provides the most accurate and generalizable age predictions for diverse biological applications.

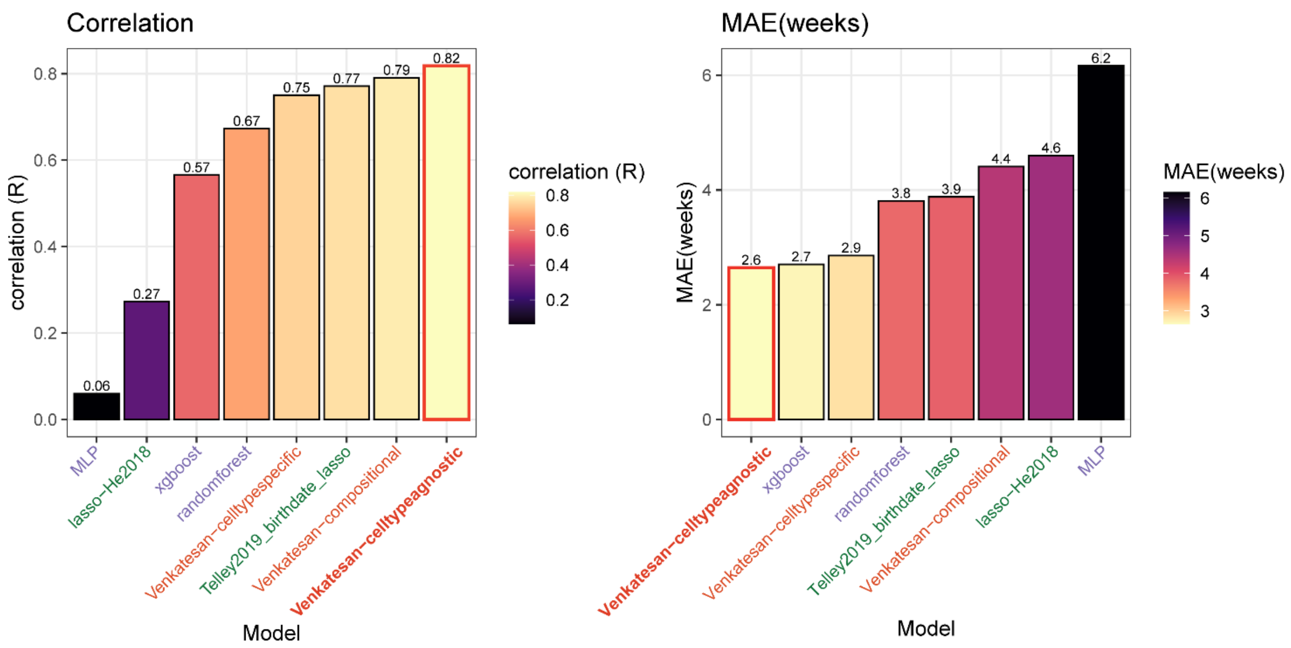


**Supplementary Figure 8: Summary comparison of all models highlights cell type-agnostic model as top performer.** Plots show median correlation and mean absolute error (MAE) for each of the models on the x-axis. Venkatesan-celltypeagnostic, Venkatesan-celltypespecific, and Venkatesan-compositional are models developed by us. MLP, xgboost and randomforest are more complex models trained on our data. Lasso-He2018 and Telley2018_birthdate_lasso are models trained on our data but using gene sets derived from [1,3] as external comparisons.

5. In the analysis identifying astrocyte proportions as predictive of developmental age, did the authors perform multiple hypothesis test correction (Fig. 2D, 2F)? The performance improvement does not appear very significant and could reflect multiple testing across many cell types.

We thank the reviewer for raising the question of multiple hypothesis test correction for the analysis involving cell type proportion-based age prediction. Our initial analysis identified astrocyte-progenitor proportions as the best predictive feature based on ranked performance metrics. We now compare model performances to a baseline model trained using scrambled data (randomly permuted ages). For models using single cell types (**Fig. 2D**), only the Progenitor model shows significantly higher performance as measured by both MAE (Wilcoxon P = 0.014, FDR adjusted P = 0.099), and correlation (P = 0.007, FDR adjusted P = 0.024). Similarly, for combinations of two cell types (**Fig. 2F**), only the Astrocyte-Progenitor model shows significantly higher performance compared to baseline by both MAE (P = 0.006, FDR adjusted P = 0.12) and correlation between predicted and actual ages (P = 0.004, FDR adjusted P = 0.018).

**Reviewer #3: This manuscript develops several statistical models to predict single-cell and tissue transcriptomic ages during early development. The predicted ages can serve as a clock to assess developmental timings across different studies, which is of significant interest to the community. The authors integrated multiple single-cell RNA-seq datasets, including 2.8 million cells, and validated their predictions using different datasets. Notably, they created a cell-type-agnostic model that does not require cell type information to estimate transcriptomic age for entire single-cell datasets. The manuscript also applies these models to organoid data and further examines mouse embryonic development. Overall, the study was logically designed from tissue to single-cell modeling. I have several comments on the methodological rigor, biological significance, and usage.**

We thank the reviewer for their insightful comments that helped us improve methodological rigor and better explain the biological significance of our findings.

1. It is unclear what glmnet regularization(s) the cell-type-agnostic model uses.

We apologize for the lack of clarity. The cell-type-agnostic model uses **elastic net regularization** implemented in glmnet. Both the mixing parameter **α** and the regularization strength **λ** were selected by cross-validation to minimize mean absolute error on the training data. For the final model reported in the manuscript, the optimal values were **α = 0.1** and **λ = 0.3377**, corresponding to a ridge-regularized elastic net model with a modest L1 component. These details are now explicitly stated in the Methods section.

2. The cell-type-specific models are insufficiently described. How many genes did they input, all 10k genes like agnostic model or cell type marker genes? It is a surprise that Fig 3 shows a lack of cell type specificity if cell type marker genes were used.

We thank the reviewer for requesting clarification and apologize for the lack of detail in the previous version. The cell-type-specific models were trained using the same full set of 10,957 genes as the cell-type-agnostic model, rather than restricting input to known marker genes.

As noted in our response to Reviewer 1 (point 5), across all cell type-specific models, mRNA binding m6A reader proteins ***IGF2BP1* and *IGF2BP2*** consistently emerge among the top features, indicating that shared RNA regulatory programs specify temporal progression across all lineages. In addition, we also find established cell-type-specific markers among top features used by the cell type-specific models (**Supplementary Fig. 10)**, such as transcription factors *NFIX* and *NFIC* in progenitor cells; *CAMK2B* in excitatory neurons; glutamate transporter *SLC7A11* in astrocytes; *MVP* and *STOM* in vascular cells etc. GO enrichment of the top features per model confirms cell type-specific terms including cell cycle, glial cell migration, glutamate transport, and mesoderm morphogenesis. These results parallel our GO term-based models (**Fig 6**) which show strong cell type-specific performance driven by canonical gene sets such as cell cycle, cell-cell adhesion, and Wnt signaling.

The apparent lack of strict cell-type specificity in **Fig. 3** highlights the coexistence of shared temporal regulators across lineages as well as lineage-specific programs within the cell-type-specific models.

3. Also, did the cell-type spec model use glmnet, the same regularized model as the agnostic one?

Yes. All cell-type-specific models were trained using the same elastic net regression framework (glmnet) as the cell-type-agnostic model, with hyperparameters selected by cross-validation within each model.

4. How conserved (or spec) are co-expression modules of 462 cell-type agnostic genes across different. studies? Is it also possible to calculate/assess the modular ages beyond individual genes/cells, which may provide additional insights into functional/mechanistic aging?

We thank the reviewer for this insightful question. We previously identified 20 co-expression modules of cell type-agnostic model genes using aggregate networks built from all studies. These modules show dynamic age-dependent expression trends, with positive modules increasing and negative modules decreasing in expression at distinct rates (**Supplementary Fig. 11**).

To asses module conservation within individual studies, we examined co-expression strength of each module within each study using EGAD [41]. All modules show robust co-expression within each study, with mean EGAD AUROC values ranging from 0.71 to 0.91. Importantly, comparing module co-expression strength across studies reveals clear clustering by age, with studies grouping together by trimester (**Supplementary Fig. 11C**). Correlation-based clustering of studies based on module co-expression identifies four major groups that clearly separate based on age, indicating that modules track temporal milestones in fetal brain development.

To examine whether module co-expression reflects conserved biology, we assessed cross-species co-expression conservation of model genes using meta-analytic RNA-seq networks from CoCoCoNET [42]. Compared to random gene sets matched for size, the 462 model genes show significantly higher conservation of co-expression across a wide-range of mammals (**Supplementary Fig. 12A,** Empirical p-values: Chimpanzee = 2e-04; Rhesus macaque = 1e-04; Crab-eating macaque = 0.0027; Mouse, Rat = 1e-04; Rabbit = 0.0217; Dog = 0.0024; Boar = 1e-04; Goat = 0.0249; Cow = 8e-04; Horse = 1e-04).

Together, these analyses demonstrate that the cell type-agnostic model genes form robust, conserved co-expression modules that track temporal milestones of human brain development, and are conserved across mammalian species.

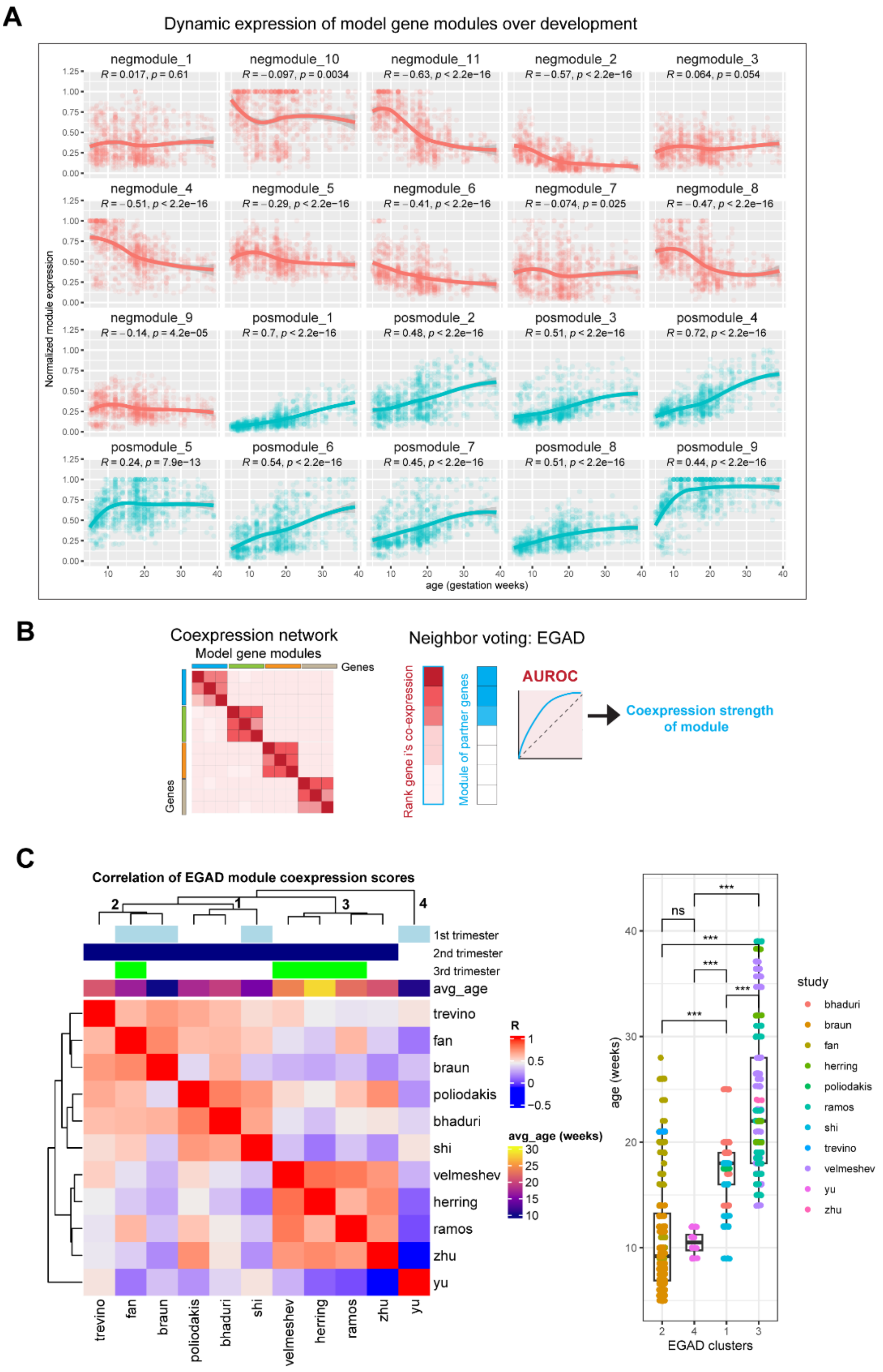
A
Dynamic expression of model gene modules over development
negmodule_1
R = 0.017, p = 0.61
negmodule_10
R = −0.097, p = 0.0034
negmodule_11
R = −0.63, p < 2.2e−16
negmodule_2
R = −0.57, p < 2.2e−16
negmodule_3
R = 0.064, p = 0.054
negmodule_4
R = −0.51, p < 2.2e−16
negmodule_5
R = −0.29, p < 2.2e−16
negmodule_6
R = −0.41, p < 2.2e−16
negmodule_7
R = −0.074, p = 0.025
negmodule_8
R = −0.47, p < 2.2e−16
negmodule_9
R = −0.14, p = 4.2e−05
posmodule_1
R = 0.7, p < 2.2e−16
posmodule_2
R = 0.48, p < 2.2e−16
posmodule_3
R = 0.51, p < 2.2e−16
posmodule_4
R = 0.72, p < 2.2e−16
posmodule_5
R = 0.24, p = 7.9e−13
posmodule_6
R = 0.54, p < 2.2e−16
posmodule_7
R = 0.45, p < 2.2e−16
posmodule_8
R = 0.51, p < 2.2e−16
posmodule_9
R = 0.44, p < 2.2e−16
Normalized module expression
age (gestation weeks)
B
Coexpression network
Model gene modules
Genes
Genes
Neighbor voting: EGAD
Rank gene i's co-expression
Module of partner genes
AUROC
Coexpression strength of module
C
Correlation of EGAD module coexpression scores
2
1
3
4
1st trimester
2nd trimester
3rd trimester
avg_age
trevino
fan
braun
poliodakis
bhaduri
shi
velmeshev
herring
ramos
zhu
yu
R
1
0.5
0
−0.5
avg_age (weeks)
30
25
20
15
10
ns
***
age (weeks)
EGAD clusters
study
bhaduri
braun
fan
herring
poliodakis
ramos
shi
trevino
velmeshev
yu
zhu

**Supplementary Figure 11. Co-expression modules from model genes show dynamic expression and track development.**
**A,** Dynamic expression trends of each module from the 462 cell type-agnostic model genes. Plots show normalized average expression of genes per module. **B,** Schematic of EGAD pipeline to determine co-expression strength of a gene set. **C,** Correlation of module EGAD scores between studies identifies grouping by developmental time. Studies sampling similar time points group together.

5. Moreover, how do those 462 genes relate to tissue PCs if they are free to cell types?

We appreciate the reviewer's interest in connecting cell-autonomous transcriptomic signatures to tissue-level principal components (PCs). We assessed whether the 462 cell-type-agnostic model genes are enriched among genes with large loadings for PCs that correlate with age within each study. If model genes are at the top of the ranked PC loadings, the AUROC will be close to 1, and 0.5 if there is no signal. For positive model genes, the highest AUROC is in the Ramos dataset (0.74) while other studies range from 0.45 to 0.6 (Mean = 0.57). Vice versa, for negative model genes, none of the datasets show AUROC > 0.6, with values ranging from 0.4 to 0.58 (Mean = 0.48). These results indicate only weak overlap between the model genes and the tissue PCs, consistent with the fact that the cell type-agnostic model leverages cell autonomous transcriptomic signals that are largely orthogonal to tissue-level variation, which is influenced by cell-type composition and technical effects.

6. Is it possible to comment on how a cell-type agnostic model may help identify novel cell types and aging?

We thank the reviewer for raising this interesting point. A cell-type-agnostic model can help identify novel or vulnerable cell populations by providing a cell-intrinsic temporal reference against which deviations can be detected. In large-scale atlases, cells or subpopulations whose ages systematically diverge from the expected trajectory for their annotated lineage may represent previously unrecognized cell states or transitional populations.

We already demonstrate this principle in the context of neurodevelopment, where the model identifies lineage-specific maturation differences in GABAergic neurons in organoids carrying ASD-associated mutations. Although the current model is trained on developmental rather than aging data, the same framework could be extended to later life stages to identify cell types that show accelerated or delayed aging relative to others. Exploring the relationship between developmental timing programs and aging-related transcriptional changes is an interesting direction for future work but is beyond the scope of the present study.

7. The prediction for organoids has relatively large ranges (weeks of variation, Fig. 7)

The variance in predicted ages for the organoids in Fig. 7 encompasses both inter-batch and inter-cell type variability. Of note, similar biological variability in predicted ages exist in the fetal data as well. To quantify variability explicitly, we computed within-sample dispersion of predicted developmental age (SD) conditioned on observed age. We find that the magnitude of within-sample variability in organoids (1.57 weeks) is comparable to that observed in primary fetal

samples (2.08 weeks). This indicates that the spread of predicted ages in organoids reflects intrinsic biological heterogeneity that is already present in developing brain tissue, rather than instability of the model.

8. Some performance comparisons need further justifications for rigor, such as 30.6% astrocytes for GO term models, R=-0.38 w/o significance, 3-week cutoff for AUROC in Fig. 7, etc.

The percentages reported (e.g., 30.6% in astrocytes) are descriptive outcomes, representing the fraction of GO term–restricted models that outperform the corresponding cell-type-specific model trained on the full gene set; they are not thresholds or tuned parameters. We have revised the text to make this explicit. Importantly, regardless of cell type, no individual GO term model outperformed the cell-type-agnostic model trained on all genes.

Both the reported correlation coefficients in Fig. 7B (R = -0.56, and R = −0.38) are statistically significant (P < 2.2e-16) and we have revised the figure legend and text to reflect this. We have also clarified that Fig. 7C reports AUROC values computed across a broad range of age-difference thresholds (1–15 weeks), with the 3-week window described in the text as an illustrative example rather than an optimized or fixed cutoff. These clarifications have been incorporated into the revised manuscript and figure legends.

9. In the tissue modeling, it is unclear how sensitive cell class/type annotation can affect the age prediction via cell compositions. Additionally, the tissue-regularized regression models should be elaborated and compared with cell-type models.

We appreciate the reviewer's concern about relying on the author-provided metadata to predict age via cell type compositions. To address this, we evaluated cell class replicability using MetaNeighbor [43]. Grouping author provided cell types into 7 major classes, we assessed whether cell types are replicable across all 9 fetal brain datasets used in training. Major cell types are highly replicable across all studies (mean AUROC: Astrocyte = 0.95, Neuron = 0.80, Immune = 0.86, Oligo = 0.90, Progenitor = 0.84, Vascular = 0.87), with lower replicability only for the "Other" category (0.63) which is a heterogeneous mix of cell types. In a more stringent one-vs-next-best comparison, we observe consistent discrimination between closely related classes (mean AUROC = 0.68), with cells clustering by cell class rather than by study. These results (**Supplementary Fig. 2**) indicate that cell-class annotations are sufficiently robust at the resolution used for tissue-composition modeling.

The tissue-level regression models are trained exclusively on cell-type proportions per sample and reflect a coarser biological signal which is more susceptible to study differences. The Astrocyte-Progenitor composition model provides coarser resolution (~4 weeks) which fails to discriminate organoids close in culture age at very early time points. In contrast, the transcriptome-based cell-type agnostic model achieves higher temporal resolution (~2 weeks) and can detect subtle maturational differences between experimental conditions, correctly discriminating nearly all adjacent organoid ages (AUROC = 1 for all but one comparisons). **Supplementary Fig. 8** provides a comprehensive comparison of performance metrics for all models used in our study.

Importantly, despite differences in the features used and their resolution, both tissue and transcriptome-based models show strong agreement in developmental trends: **Fig. 8B** demonstrates high correlation between average predicted ages from tissue-composition and

transcriptome-based cell type-agnostic models across systems (human: R = 0.69, P < 2.2e-16, mouse: R = 0.86, P < 2.2e-16, human neural organoids: R = 0.94, P = 4.8e-13).

**Reviewer #4: In this manuscript, Venkatesan et al. present a large-scale meta-analysis of 13 human fetal brain scRNA-seq datasets (~2.9 million cells) aimed at disentangling tissue-level composition effects from cell-autonomous maturation and at developing broadly generalizable developmental age predictors. The authors show that study-specific composition and PC-based age proxies perform well within individual datasets but fail to generalize across studies, largely because PCs capture shifts in cell type proportions. To address this, they derive a remarkably simple and transferable compositional model based on the relative increase of astrocytes and decrease of progenitors, and complement it with a regularized, cell type-agnostic transcriptomic model that reliably predicts age across cell types, datasets, organoids, and even mouse embryonic brain. Together with the freely available code, these models provide a practical framework for aligning neurodevelopmental tempo across systems.**

We thank the reviewer for their appreciation of our method and their suggestions to test interesting biological applications of our approach.

1) A key question is how many datasets are minimally required to achieve robust performance. A subsampling analysis using the existing 13 datasets would be informative, for instance by training region-specific models (e.g. a cortex-dominant model) and comparing their performance against the full model on held-out non-cortical datasets, such as the hypothalamus data in Figure 3E. This would directly test whether the model's generalizability depends on the anatomical diversity of the training set.

5) The manuscript would benefit from a more comprehensive analysis of prediction errors across developmental time. Reporting only median errors masks potential biases. Stratifying the dataset into early, mid, and late developmental stages and recalculating prediction errors for each would reveal whether accuracy is uniform across development or biased toward particular stages.

We appreciate the reviewer's request to quantify (i) how many datasets are required to achieve robust performance, (ii) how prediction accuracy varies across developmental time, and (iii) whether model generalizability depends on anatomical diversity of the training set. We address these questions through a series of subsampling and stratified analyses.

**Minimum number of datasets for robust performance.**
We retrained the model on all combinations of studies using subsets ranging from 2 to 13 datasets and evaluated performance on the remaining held-out studies. This combinatorial benchmark reveals that prediction accuracy improves as more studies are included in training, but the gains plateau after approximately **6–7 studies**, indicating that the model reaches a stable regime of generalization well before all 13 datasets are used **(Supplementary Fig. 6B**).

**Prediction error across developmental time.**
Stratifying test data into first, second, and third trimester samples reveals non-uniform accuracy across development, with highest error in third-trimester samples. This bias is largely attributable to uneven sampling of developmental windows, particularly the scarcity of third-

trimester cells across datasets. Importantly, when models are trained on subsets with comparable numbers of cells from all three trimesters, prediction accuracy becomes similar across stages (mean error ≈ 5 weeks), indicating that the observed bias reflects data availability rather than model failure.

**Dependence on anatomical diversity of the training set.**
To directly test whether generalizability depends on anatomical coverage, we grouped training study combinations into **cortex-dominant** and **hypothalamus-dominant** sets and evaluated performance on held-out brain regions (**Supplementary Fig. 6C**). For cortical test data, prediction accuracy does not differ significantly between models trained with or without cortical datasets, regardless of the number of studies. This likely reflects shared cell-autonomous developmental programs across closely related forebrain regions such as the cortex and ganglionic eminences. In contrast, prediction accuracy for hypothalamic samples is reduced when no hypothalamic data are included in training. This regional dependency diminishes as additional studies are incorporated, with comparable performance between models trained with and without hypothalamic data only when ~ 9 studies are used for training.

Together, these analyses show that robust performance can be achieved with a moderate number of diverse datasets, that apparent stage-specific biases largely reflect uneven sampling across development, and that anatomical diversity in the training set improves generalizability, particularly for more specialized brain regions such as the hypothalamus.

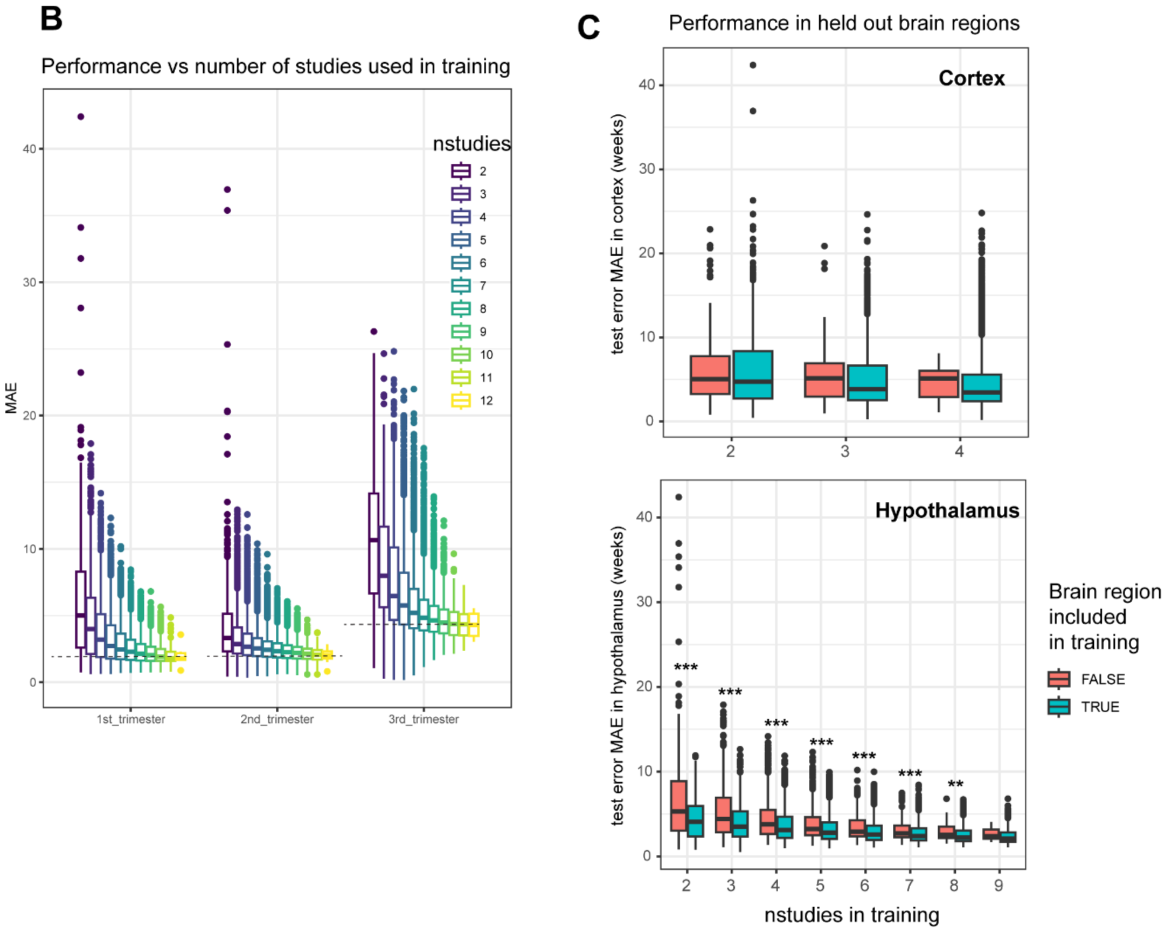


**Supplementary Fig. 6B-C. B,** Distribution of prediction error for models trained on different combinations of studies (N = 2 to 12). Performance increases as more datasets are used for training, but plateaus after ~ 7 studies. Third trimester samples show highest prediction error due to scarcity of training data. **C,** Distribution of prediction error in test datasets from cortex (top) or hypothalamus (bottom) when these brain regions are included or excluded from training data. ***P < 0.001, ** P < 0.01 (FDR adjusted Wilcoxon p-value)

2) Several of the reported differences appear to be within the variance or error range of the analysis approach. All such comparisons should be supported with appropriate statistics, including effect sizes and p-values. Since the models generally have an error of a few weeks, it would also be helpful for the authors to discuss under which circumstances this level of accuracy is sufficient and in which contexts it may be too coarse.

We appreciate the reviewer's request for clear statistical reporting. We have clarified some of the issues mentioned here in our responses to reviewer #2 and have revised the manuscript to ensure that all performance comparisons are supported by appropriate statistical tests, effect sizes, and p-values. We now include a unified summary comparison **(Supplementary Fig. 8)** reporting consistent performance metrics (median MAE and Spearman correlation) for all models evaluated, including transcriptome-based, tissue-composition, nonlinear, and baseline models. Across all approaches, the cell-type-agnostic model achieves the lowest error and highest correlation between predicted and actual age.

Transcriptome-based cell autonomous models are suitable for fine-grained developmental staging and achieve higher accuracy (~2 weeks) to detect subtle maturational differences between experimental conditions. For example, the cell type-agnostic model correctly discriminates nearly all organoid batches from different culture time points (AUROC = 1 for all but one comparisons). In contrast, the Astrocyte-Progenitor composition model provides coarser developmental resolution (error = 4.5 weeks) and fails to discriminate organoids close in culture age at very early time points (AUROCs ~ 0.25–0.38 for early developmental stages).

Given its higher accuracy, robustness across datasets, and flexibility in applications where precise cell type matching is not required, we recommend the cell type-agnostic transcriptome model as the core model for downstream analyses.

3) The authors note that organoids tend to display greater variability than primary tissues. It would strengthen the study to quantify this explicitly at the level of developmental age predictions.

To directly quantify variability at the level of developmental age predictions in organoids, we computed within-sample dispersion of predicted developmental age (SD) conditioned on observed age. The magnitude of within-sample variability in organoids (1.57 weeks) is comparable to that observed in primary fetal samples (2.08 weeks). This indicates that the spread of predicted ages in organoids reflects intrinsic biological heterogeneity that is already present in developing brain tissue, rather than instability of the model.

To further contextualize this variability, we examined co-expression of developmental age-prediction gene modules across diverse organoid datasets from [51] and compared these values to the co-expression of previously established MetaMarker gene sets defining fetal cell types [44]. Age-module co-expression AUROCs range from 0.55 to 0.75 across 24 organoid datasets comprising 173 individual organoid batches, indicating substantial variability across organoid preparations. Importantly, age-module co-expression is generally lower than cell-type MetaMarker co-expression, and the two measures are strongly correlated ($R = 0.72$, $P < 2.2e-16$), suggesting that variability in developmental age signals is coupled to broader variability in transcriptional organization.

These results are now summarized in **Supplementary Fig. 12B** which compares variability of age-module co-expression to co-expression of established cell-type transcriptional programs in diverse organoid datasets.

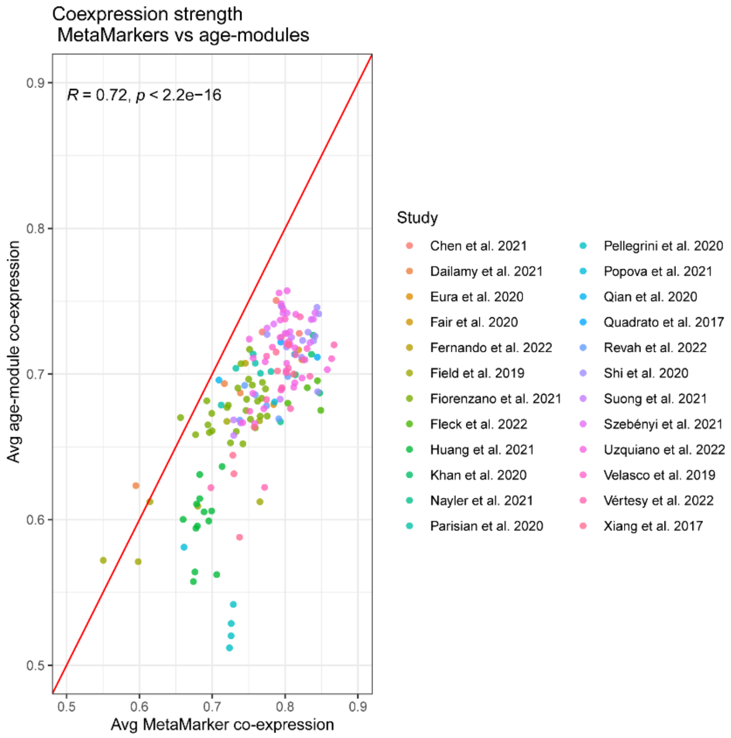


**Supplementary Fig. 12B. Variability in organoid co-expression of developmental and cell type programs**

Correlation of age prediction module co-expression scores to fetal cell type MetaMarker co-expression. Age modules show generally lower co-expression than cell type modules, but both are tightly correlated across 24 organoid datasets.

4) Many of the models plateau at older developmental ages (e.g., Fig. 1E, Fig. 3D+E, Supplementary Fig. 7A). For organoids (Supplementary Fig. 7B), the authors attribute this to metabolic constraints, yet similar flattening is apparent in the fetal brain data. Additional analyses or discussion should clarify whether this reflects biological features, limited sample availability, or computational artefacts.

We thank the reviewer for raising this important point. To determine whether the apparent plateauing of predicted developmental age reflects biological constraints, limited training data, or model artifacts, we retrained a new cell type-agnostic model that extends the training set beyond fetal development by including postnatal human brain data up to adulthood from the Velmeshev, Ramos, and Zhu datasets. This ensures that model predictions are not constrained by the upper bound of fetal ages in the original training data.

Applying this expanded model to **in vitro organoid datasets shows no change in predicted ages** compared to the fetal-only model. Specifically, predicted ages continue to plateau at 5–6 months in Uzquiano et al. organoids (Wilcoxon test comparing old and new predictions, FDR-adjusted P = 0.47) and at 3–4 months in Fiorenzano et al. organoids (P = 0.80). This indicates that the observed flattening in organoids does not arise from limitations of the training data or saturation of the regression model.

In contrast, when applied to **in vivo transplanted organoids** from Revah et al., inclusion of postnatal training data significantly increases predicted ages only for the transplanted condition. Median predicted age increases from 32 weeks with the fetal-only model to 77 weeks (~9 months postnatal) with the expanded model ($P = 2.32 \times 10^{-8}$). This selective effect demonstrates that the plateauing observed in vitro is not a computational artifact, but instead

reflects biological constraints imposed by in vitro culture conditions, which are alleviated by in vivo transplantation.

Together, these results indicate that flattening of predicted age in organoids at later time points reflects **biological limitations in maturation under in vitro conditions**, rather than restricted sample availability or model saturation. These analyses are now included in **Supplementary Fig. 15** and described in the main manuscript.

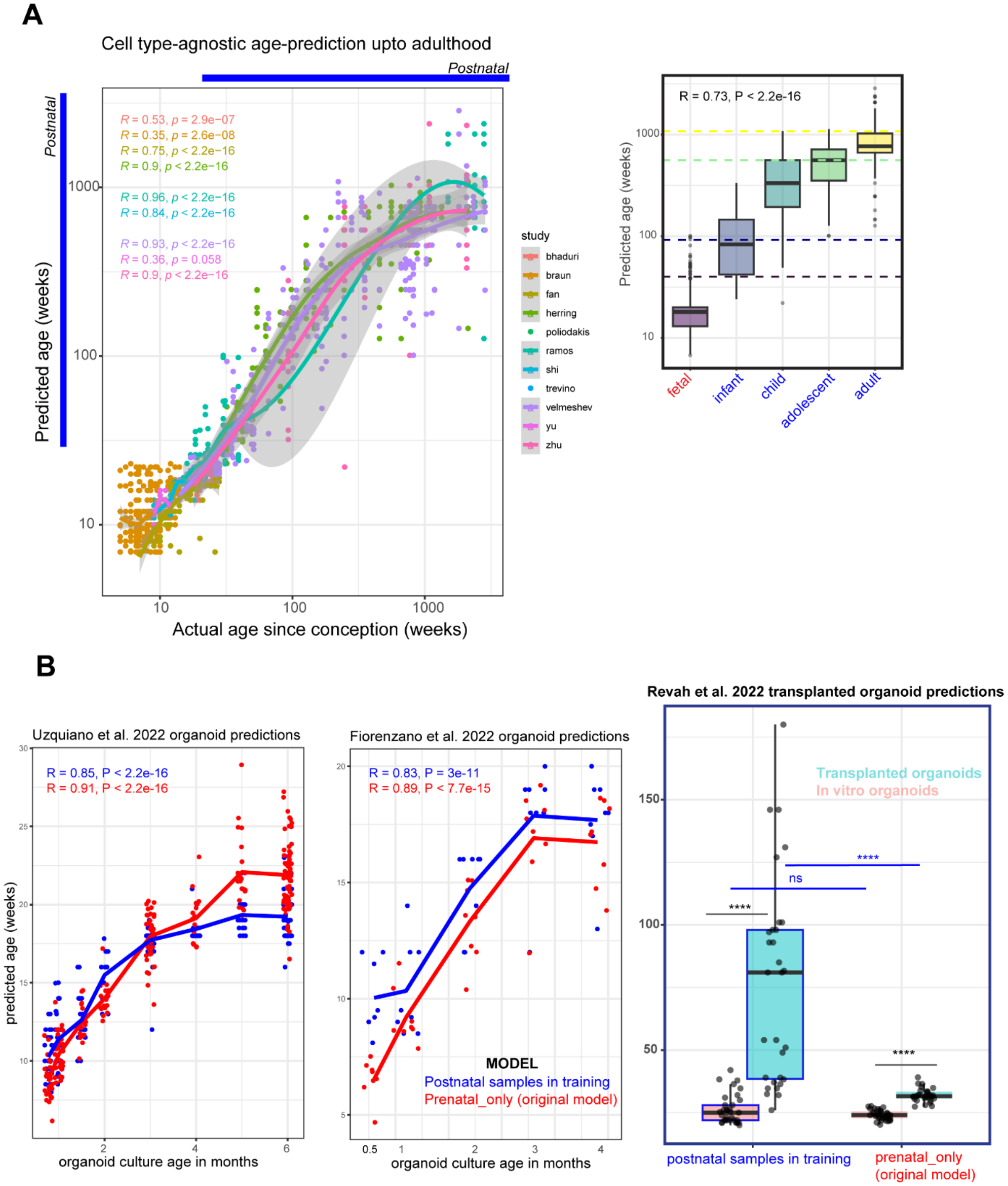

**Supplementary Figure 15: Including postnatal data in model training extends age predictions only for transplanted organoids. A,** Cross-validation performance of cell type-agnostic age prediction model trained on both prenatal and postnatal data from 11 datasets. Predicted ages are strongly correlated with actual age (weeks since conception). Boxplots show predicted ages grouped by developmental windows: prenatal (<40 gestation weeks), infancy (< 1year postnal), childhood (<10 years), adolescence (<20 years) and adulthood (> 20 years). **B,** Developmental age predictions for neural organoids using the original cell-type-agnostic model trained on prenatal data only (red) and the updated model trained on prenatal plus postnatal data (blue). In vitro organoids show a plateau in predicted age at approximately 20 gestational weeks with both models, whereas transplanted organoids exhibit substantially extended maturation with the updated model, reaching predicted ages of up to 1–2 years postnatal (****P $< 10^{-4}$, ns: not significant, FDR adjusted Wilcoxon P-value)

6) The elegant linear mapping between mouse embryonic days and human gestational weeks calls for sensitivity analyses. Testing alternative ortholog sets and excluding lineage-biased gene modules would help determine whether cross-species tempo estimates are robust or disproportionately driven by specific gene groups.

We appreciate this insightful question from the reviewer to test alternative ortholog sets and exclude lineage-biased gene modules for cross-species applications to test robustness of tempo differences to gene set choice. We address this in 3 ways:

**I. Orthogroup-based ortholog definitions**
To test alternative ortholog sets, we adopted a gene-family (orthogroup) approach using the hierarchical orthology catalog from OrthoDB. We trained age-prediction models on human data using three different feature definitions:
(1) one-to-one orthologs only;
(2) all orthogroups (including one-to-one and many-to-many orthologs, with expression averaged at the orthogroup level); and
(3) orthogroups containing multiple genes only (i.e. many-to-many orthologs).

Cross-validation performance in human data is comparable across these approaches (median MAE= 2.58, 2.78, 3.15 weeks; correlation R = 0.85, 0.75, 0.74 respectively for one2one orthologs, Orthogroups, and N2M Orthogroups). All three models accurately predict developmental progression across neural cell types in the Linarsson et al. mouse dataset (Correlation between predicted human age and actual mouse age R = 0.77,0.77,0.82). Importantly, the estimated slopes used to infer relative developmental tempo in mouse are highly consistent across ortholog definitions (95% CI for slope: [10.0-11.2], [9.3-10.4], and [8.7-9.5], respectively). Thus, the inferred ~10-fold acceleration of mouse developmental tempo relative to human is robust to ortholog-set choice.

**II) Excluding linage-biased gene modules via random subsampling**

To further test whether specific gene modules disproportionately drive tempo estimates, we trained 20 independent models, each restricted to a random subset of 1,000 one-to-one human-mouse orthologs. All models perform well in human data (median MAE = 2.6-3.7 weeks; R = 0.59-0.77) and generalize to mouse data (R = 0.47-0.77). The estimated tempo slopes range from 4.7- to 11-fold, with a mean slope of 7.84 ± 0.55 across models. Based on these results,

we now report a more conservative, bounded estimate of human–mouse developmental tempo differences in the revised manuscript.

**III) Testing synapse-biased gene sets**

Finally, we explicitly tested whether synapse-related genes bias tempo estimation by training models either exclusively on SynGO genes (1,192 genes) or on one-to-one orthologs excluding SynGO genes (8,905 genes). Human cross-validation performance does not differ significantly between these models (Wilcoxon $P = 0.243$). However, in mouse data, the SynGO-restricted model yields a modestly lower tempo slope (95% CI: [8.4–9.6]) compared to the model excluding SynGO genes (95% CI: [10.6–11.8]). These results demonstrate that gene-set choice can influence the absolute slope estimate, but even lineage-biased or narrowly defined gene sets consistently support a substantial acceleration of mouse developmental tempo (~8–9-fold) relative to human.

7) The finding of a tenfold faster developmental tempo in mice compared to humans appears strikingly high, especially relative to previous estimates of about two- to fivefold acceleration (e.g. Rayon et al. 2020, Iwata et al. 2023). Additional evidence supporting this larger factor should be cited or the claim more cautiously framed.

We thank the reviewer for highlighting the need to contextualize our estimate of a ~10-fold acceleration in mouse developmental tempo relative to humans. While estimates of species differences in developmental rate vary depending on the process and timescale considered [45–48], the general concept of significantly faster developmental progression in rodents compared to primates is well established in the literature.

Previous studies reporting smaller tempo differences (2-5×) have focused on specific molecular or cellular programs in controlled in vitro systems. For example, Rayon et al 2020 examined differentiation of embryonic stem cells into spinal motor neurons and reported a 2-3 fold difference in protein stability and developmental tempo between human and mouse. Matsuda et al. 2020 measured segmentation clock dynamics in *in vitro* iPSC derived presomitic mesoderm cells and found 2-3 x faster speed in mouse. Iwata et al. (2023) focused on mitochondrial activity and maturation in PSC-derived cortical neurons, again identifying accelerated dynamics in mouse. In contrast, xenotransplantation studies such as Linaro et al. (2019) have demonstrated a much slower intrinsic pace of neuronal morphological and electrophysiological maturation in humans, lasting ~11 months compared to ~1 month in mouse.

Our estimate of a ~10-fold difference arises from a fundamentally different approach: a data-driven analysis of transcriptomic progression across the entire neurodevelopmental timeline, rather than a single regulatory circuit, cell type, or developmental milestone. As such, our result does not contradict prior work but extends it by quantifying global developmental timing across species using large single-cell transcriptomic compendia. We have revised the manuscript to frame this estimate more cautiously and to emphasize that the magnitude of cross-species tempo differences depends on the temporal scope and biological context of the comparison.

8) To test the limits of generalizability, it would be valuable to develop a parallel cell type-agnostic model trained exclusively on mouse brain development and to compare its predictions with those of the human-trained model on the same mouse datasets. This would provide an internal benchmark for cross-species applicability and could refine the estimates of relative tempo between species.

We thank the reviewer for suggesting a parallel mouse-trained cell-type-agnostic model as an internal benchmark. We trained a meta-analytic cell type agnostic age prediction model on embryonic mouse brain data (N =10 datasets) [49–58] and evaluated it alongside the human-trained model across species. In both directions, predicted ages are strongly correlated with chronological age (R = 0.93, 0.85 for mouse model on mouse and human data respectively; and R = 0.87, 0.54 for human model on human and mouse data respectively), demonstrating that conserved cell-autonomous transcriptional programs preserve developmental ordering across species.

When applying the human-trained model to mouse datasets, the inferred slopes are consistently greater than one (upper limit ~3.9 - 8.4 across different mouse datasets), indicating faster progression of mouse development along a shared transcriptomic axis. In contrast, applying the mouse-trained model to human datasets yields smaller slopes (≈ 0.02–0.07), consistent with slower human maturation. Notably, these slopes are not expected to be direct reciprocals of the human to mouse mapping, as the regressions involve different dynamic ranges (training data spans 40 weeks in human, but 20 days in mouse) and non-zero intercepts which introduce scale compression in cross-species projection.

We therefore interpret the mouse-trained model as an internal benchmark for cross-species ordering rather than absolute calibration, and base tempo estimates on the direction that preserves dynamic range and interpretability (human to mouse). We report these results in our revised manuscript and have framed our cross-species tempo estimates more cautiously.

9) On page 4, line 137, the authors mention two coefficients. These should be directly referenced to the corresponding tables or figures where the values are presented to aid clarity.

We thank the reviewer for bringing this to our notice, we have updated the coefficients in Fig. 2E where the Astrocyte-Progenitor model is used.

10) Finally, while fully optional, a direct experimental test in human neural organoids would be highly compelling. For example, subjecting organoids at a fixed developmental stage to a defined stressor such as hypoxia and then measuring the predicted age shift relative to controls would provide strong evidence that the model captures state-dependent changes independent of chronological time.

We thank the reviewer for suggesting this optional experiment to further demonstrate state-dependent sensitivity of the model. We identified a recently published human neural organoid dataset in which organoids at matched culture ages were subjected to hypoxic versus hyperoxic conditions and profiled by single-cell RNA sequencing [4]. Applying our cell type-agnostic age prediction model to this dataset reveals that hypoxic organoids exhibit significantly delayed developmental progression relative to controls, despite identical chronological culture time (mean difference in predicted age = 1.2 weeks, FDR-adjusted Wilcoxon test P = 0.035). This result provides a direct experimental demonstration that the model captures state-dependent shifts in developmental maturity independent of chronological time. These findings are shown in **Supplementary Fig. 16A**.

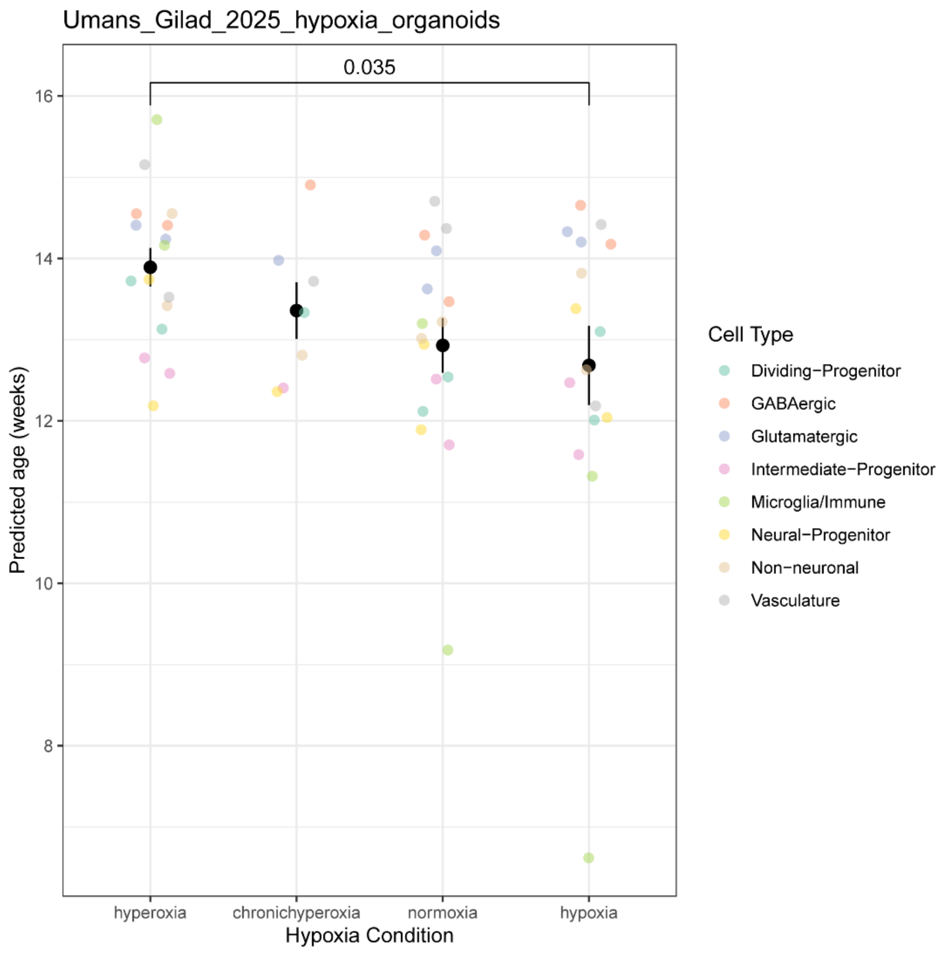


**Supplementary Figure 16A: Developmental age predictions in hypoxic human neural organoids from Umans and Gilad 2025.** Cell type-agnostic age predictions show significant delay in maturation in neural organoids grown in hypoxia compared to hyperoxia.

## References


1. He Z, Yu Q. Identification and characterization of functional modules reflecting transcriptome transition during human neuron maturation. BMC Genomics. 2018;19: 262. doi:10.1186/s12864-018-4649-2

2. Darmanis S, Sloan SA, Zhang Y, Enge M, Caneda C, Shuer LM, et al. A survey of human brain transcriptome diversity at the single cell level. Proc Natl Acad Sci U S A. 2015;112: 7285–7290. doi:10.1073/pnas.1507125112

3. Telley L, Agirman G, Prados J, Amberg N, Fièvre S, Oberst P, et al. Temporal patterning of apical progenitors and their daughter neurons in the developing neocortex. Science. 2019;364: eaav2522. doi:10.1126/science.aav2522

4. Umans BD, Gilad Y. Oxygen-induced stress reveals context-specific gene regulatory effects in human brain organoids. Genome Res. 2025;35: 1689–1700. doi:10.1101/gr.280219.124

5. Nolbrant S, Wallace JL, Ding JW, Zhu T, Sevetson JL, Kajtez J, et al. Interspecies Organoids Reveal Human-Specific Molecular Features of Dopaminergic Neuron Development and Vulnerability. bioRxiv; 2025. p. 2024.11.14.623592. doi:10.1101/2024.11.14.623592

6. Liu Y, Cai F, Barile M, Chang Y, Cao D, Huang Y. TemporalVAE: atlas-assisted temporal mapping of time-series single-cell transcriptomes during embryogenesis. Nat Cell Biol. 2025;27: 1982–1992. doi:10.1038/s41556-025-01787-7

7. Breiman L. Random Forests. Mach Learn. 2001;45: 5–32. doi:10.1023/A:1010933404324

8. Fisher A, Rudin C, Dominici F. All Models are Wrong, but Many are Useful: Learning a Variable's Importance by Studying an Entire Class of Prediction Models Simultaneously. arXiv; 2019. doi:10.48550/arXiv.1801.01489

9. Yoon K-J, Ringeling FR, Vissers C, Jacob F, Pokrass M, Jimenez-Cyrus D, et al. Temporal Control of Mammalian Cortical Neurogenesis by m6A Methylation. Cell. 2017;171: 877-889.e17. doi:10.1016/j.cell.2017.09.003

10. Klein P, Petrić Howe M, Harley J, Crook H, Esteban Serna S, Roumeliotis TI, et al. m6a methylation orchestrates IMP1 regulation of microtubules during human neuronal differentiation. Nat Commun. 2024;15: 4819. doi:10.1038/s41467-024-49139-7

11. Gleeson J, Madugalle SU, Wan CY, McLean C, Bredy TW, De Paoli-Iseppi R, et al. Isoform-level profiling of m6A epitranscriptomic signatures in human brain. Sci Adv. 2025;11: eadp0783. doi:10.1126/sciadv.adp0783

12. Liu Z, Yang C-P, Sugino K, Fu C-C, Liu L-Y, Yao X, et al. Opposing intrinsic temporal gradients guide neural stem cell production of varied neuronal fates. Science. 2015;350: 317–320. doi:10.1126/science.aad1886

13. Lee JY, Huang N, Samuels TJ, Davis I. Imp/IGF2BP and Syp/SYNCRIP temporal RNA interactomes uncover combinatorial networks of regulators of Drosophila brain development. Sci Adv. 2025;11: eadr6682. doi:10.1126/sciadv.adr6682

14. Samuels TJ, Järvelin AI, Ish-Horowicz D, Davis I. Imp/IGF2BP levels modulate individual neural stem cell growth and division through myc mRNA stability. Desplan C, Banerjee U, Doe CQ, editors. eLife. 2020;9: e51529. doi:10.7554/eLife.51529

15. Valentino P, Islam IM, Arain U, Erclik T. Concurrent temporal patterning of neural stem cells in the fly visual system. Nat Commun. 2025;16: 8405. doi:10.1038/s41467-025-63416-z

16. Hamid A, Gattuso H, Caglar AN, Pillai M, Steele T, Gonzalez A, et al. The conserved RNA-binding protein Imp is required for the specification and function of olfactory navigation circuitry in *Drosophila*. Curr Biol. 2024;34: 473-488.e6. doi:10.1016/j.cub.2023.12.020

17. Azur RAG, Feliciano D, Espinosa-Medina I, Adhikari R, Lilao-Garzón J, Jensen E, et al. Imp1 acts as a dosage- and stage-dependent temporal rheostat orchestrating radial glial fate transitions and cortical morphogenesis. bioRxiv; 2025. p. 2025.11.18.688993. doi:10.1101/2025.11.18.688993

18. Núñez L, Buxbaum AR, Katz ZB, Lopez-Jones M, Nwokafor C, Czaplinski K, et al. Tagged actin mRNA dysregulation in IGF2BP1−/− mice. Proc Natl Acad Sci. 2022;119: e2208465119. doi:10.1073/pnas.2208465119

19. Franklin A, Davies JP, Clifton NE, Blake GET, Bamford R, Walker EM, et al. Cell-type-specific DNA methylation dynamics in the prenatal and postnatal human cortex. Cell Genomics. 2025;0. doi:10.1016/j.xgen.2025.101010

20. DiSanza BL, Porcari GS, Finoti LS, Ramos-Rodriguez L, Burris DM, McDonough JA, et al. Bi-allelic variants in BCAT1 impair mitochondrial function and are associated with a candidate neurometabolic disorder. Hum Genet Genomics Adv. 2026;7. doi:10.1016/j.xhgg.2025.100525

21. Zhang S, Zhao J, Zhao C, Su L, Jiao J. BCAT1 controls embryonic neural stem cells proliferation and differentiation in the upper layer neurons. Mol Brain. 2023;16: 53. doi:10.1186/s13041-023-01044-8

22. Lalani SR, Zhang J, Schaaf CP, Brown CW, Magoulas P, Tsai AC-H, et al. Mutations in PURA Cause Profound Neonatal Hypotonia, Seizures, and Encephalopathy in 5q31.3 Microdeletion Syndrome. Am J Hum Genet. 2014;95: 579–583. doi:10.1016/j.ajhg.2014.09.014

23. Herrero Martín JC, Salegi Ansa B, Álvarez-Rivera G, Domínguez-Zorita S, Rodríguez-Pombo P, Pérez B, et al. An ETFDH-driven metabolon supports OXPHOS efficiency in skeletal muscle by regulating coenzyme Q homeostasis. Nat Metab. 2024;6: 209–225. doi:10.1038/s42255-023-00956-y

24. Lish AM, Grogan EFL, Benoit CR, Pearse RV, Heuer SE, Luquez T, et al. CLU alleviates Alzheimer's disease-relevant processes by modulating astrocyte reactivity and microglia-dependent synaptic density. Neuron. 2025;113: 1925-1946.e11. doi:10.1016/j.neuron.2025.03.034

25. Bernhardt C, Sock E, Fröb F, Hillgärtner S, Nemer M, Wegner M. KLF9 and KLF13 transcription factors boost myelin gene expression in oligodendrocytes as partners of SOX10 and MYRF. Nucleic Acids Res. 2022;50: 11509–11528. doi:10.1093/nar/gkac953

26. Choi E-B, Vodnala M, Saini P, Anugula S, Zerbato M, Ho JJ, et al. Transcription factor SOX15 regulates stem cell pluripotency and promotes neural fate during differentiation by activating the neurogenic gene Hes5. J Biol Chem. 2023;299: 102996. doi:10.1016/j.jbc.2023.102996

27. Benoit CR, Sattler LB, Aylward AJ, Pembridge O, Kim B, Muratore CR, et al. POU3F2 regulates canonical Wnt signalling via SOX13 and ADNP to expand the neural progenitor population. Brain. 2025; awaf221. doi:10.1093/brain/awaf221

28. Kool MJ, Proietti Onori M, Borgesius NZ, van de Bree JE, Elgersma-Hooisma M, Nio E, et al. CAMK2-Dependent Signaling in Neurons Is Essential for Survival. J Neurosci. 2019;39: 5424–5439. doi:10.1523/JNEUROSCI.1341-18.2019

29. Campbell CE, Piper M, Plachez C, Yeh Y-T, Baizer JS, Osinski JM, et al. The transcription factor Nfix is essential for normal brain development. BMC Dev Biol. 2008;8: 52. doi:10.1186/1471-213X-8-52

30. Heng YHE, McLeay RC, Harvey TJ, Smith AG, Barry G, Cato K, et al. NFIX Regulates Neural Progenitor Cell Differentiation During Hippocampal Morphogenesis. Cereb Cortex N Y NY. 2014;24: 261–279. doi:10.1093/cercor/bhs307

31. Muñoz-Manchado AB, Gonzales CB, Zeisel A, Munguba H, Bekkouche B, Skene NG, et al. Diversity of Interneurons in the Dorsal Striatum Revealed by Single-Cell RNA Sequencing and PatchSeq. Cell Rep. 2018;24: 2179-2190.e7. doi:10.1016/j.celrep.2018.07.053

32. Lemieux SP, Lev-Ram V, Tsien RY, Ellisman MH. Perineuronal nets and the neuronal extracellular matrix can be imaged by genetically encoded labeling of HAPLN1 in vitro and in vivo. bioRxiv; 2023. p. 2023.11.29.569151. doi:10.1101/2023.11.29.569151

33. Shinoda Y, Sadakata T, Nakao K, Katoh-Semba R, Kinameri E, Furuya A, et al. Calcium-dependent activator protein for secretion 2 (CAPS2) promotes BDNF secretion and is critical for the development of GABAergic interneuron network. Proc Natl Acad Sci. 2011;108: 373–378. doi:10.1073/pnas.1012220108

34. Ottestad-Hansen S, Hu QX, Follin-Arbelet VV, Bentea E, Sato H, Massie A, et al. The cystine-glutamate exchanger (xCT, Slc7a11) is expressed in significant concentrations in a subpopulation of astrocytes in the mouse brain. Glia. 2018;66: 951–970. doi:10.1002/glia.23294

35. Cuellar-Santoyo AO, Ruiz-Rodríguez VM, Mares-Barbosa TB, Patrón-Soberano A, Howe AG, Portales-Pérez DP, et al. Revealing the contribution of astrocytes to glutamatergic neuronal transmission. Front Cell Neurosci. 2023;16. doi:10.3389/fncel.2022.1037641

36. Park S, Park H, Byun YG, Wei X, Eom J, Joo J, et al. NR3C1-mediated epigenetic regulation suppresses astrocytic immune responses in mice. Nat Commun. 2025;16: 8330. doi:10.1038/s41467-025-64088-5

37. Purmann C, Farrise K, Huang Y, Pattni R, Ho M, Carrion VG, et al. Effect of cortisol on cortical organoids: Building a “stress in a dish” model system. bioRxiv; 2025. p. 2025.09.16.676476. doi:10.1101/2025.09.16.676476

38. Jiang B, Bai F, Hu Y, Ren Y, Su Y, Song W, et al. Endothelial major vault protein alleviates vascular remodeling via promoting Parkin-mediated mitophagy. Nat Commun. 2025;16: 4365. doi:10.1038/s41467-025-59644-y

39. Fricke B, Argent AC, Chetty MC, Pizzey AR, Turner EJ, Ho MM, et al. The “stomatin” gene and protein in overhydrated hereditary stomatocytosis. Blood. 2003;102: 2268–2277. doi:10.1182/blood-2002-06-1705

40. Kajiwara Y, Wang E, Wang M, Sin WC, Brennand KJ, Schadt E, et al. GJA1 (connexin43) is a key regulator of Alzheimer’s disease pathogenesis. Acta Neuropathol Commun. 2018;6: 144. doi:10.1186/s40478-018-0642-x

41. Ballouz S, Weber M, Pavlidis P, Gillis J. EGAD: ultra-fast functional analysis of gene networks. Bioinformatics. 2017;33: 612–614. doi:10.1093/bioinformatics/btw695

42. Lee J, Shah M, Ballouz S, Crow M, Gillis J. CoCoCoNet: conserved and comparative co-expression across a diverse set of species. Nucleic Acids Res. 2020;48: W566–W571. doi:10.1093/nar/gkaa348

43. Crow M, Paul A, Ballouz S, Huang ZJ, Gillis J. Characterizing the replicability of cell types defined by single cell RNA-sequencing data using MetaNeighbor. Nat Commun. 2018;9: 884. doi:10.1038/s41467-018-03282-0

44. Werner JM, Gillis J. Meta-analysis of single-cell RNA sequencing co-expression in human neural organoids reveals their high variability in recapitulating primary tissue. PLOS Biol. 2024;22: e3002912. doi:10.1371/journal.pbio.3002912

45. Rayon T, Stamataki D, Perez-Carrasco R, Garcia-Perez L, Barrington C, Melchionda M, et al. Species-specific pace of development is associated with differences in protein stability. Science. 2020;369: eaba7667. doi:10.1126/science.aba7667

46. Iwata R, Casimir P, Erkol E, Boubakar L, Planque M, Gallego López IM, et al. Mitochondria metabolism sets the species-specific tempo of neuronal development. Science. 2023;0: eabn4705. doi:10.1126/science.abn4705

47. Ciceri G, Baggiolini A, Cho HS, Kshirsagar M, Benito-Kwiecinski S, Walsh RM, et al. An epigenetic barrier sets the timing of human neuronal maturation. Nature. 2024;626: 881–890. doi:10.1038/s41586-023-06984-8

48. Matsuda M, Hayashi H, Garcia-Ojalvo J, Yoshioka-Kobayashi K, Kageyama R, Yamanaka Y, et al. Species-specific segmentation clock periods are due to differential biochemical reaction speeds. Science. 2020;369: 1450–1455. doi:10.1126/science.aba7668

49. Qiu C, Martin BK, Welsh IC, Daza RM, Le T-M, Huang X, et al. A single-cell time-lapse of mouse prenatal development from gastrula to birth. Nature. 2024;626: 1084–1093. doi:10.1038/s41586-024-07069-w

50. Hamed AA, Kunz DJ, El-Hamamy I, Trinh QM, Subedar OD, Richards LM, et al. A brain precursor atlas reveals the acquisition of developmental-like states in adult cerebral tumours. Nat Commun. 2022;13: 4178. doi:10.1038/s41467-022-31408-y

51. Schmitz MT, Sandoval K, Chen CP, Mostajo-Radji MA, Seeley WW, Nowakowski TJ, et al. The development and evolution of inhibitory neurons in primate cerebrum. Nature. 2022;603: 871–877. doi:10.1038/s41586-022-04510-w

52. Butts JC, Wu S-R, Durham MA, Dhindsa RS, Revelli J-P, Ljungberg MC, et al. A single-cell transcriptomic map of the developing Atoh1 lineage identifies neural fate decisions and neuronal diversity in the hindbrain. Dev Cell. 2024;59: 2171-2188.e7. doi:10.1016/j.devcel.2024.07.007

53. Di Bella DJ, Habibi E, Stickels RR, Scalia G, Brown J, Yadollahpour P, et al. Molecular logic of cellular diversification in the mouse cerebral cortex. Nature. 2021;595: 554–559. doi:10.1038/s41586-021-03670-5

54. Loo L, Simon JM, Xing L, McCoy ES, Niehaus JK, Guo J, et al. Single-cell transcriptomic analysis of mouse neocortical development. Nat Commun. 2019;10: 134. doi:10.1038/s41467-018-08079-9

55. Govek KW, Chen S, Sgourdou P, Yao Y, Woodhouse S, Chen T, et al. Developmental trajectories of thalamic progenitors revealed by single-cell transcriptome profiling and Shh perturbation. Cell Rep. 2022;41. doi:10.1016/j.celrep.2022.111768

56. Kim DW, Washington PW, Wang ZQ, Lin SH, Sun C, Ismail BT, et al. The cellular and molecular landscape of hypothalamic patterning and differentiation from embryonic to late postnatal development. Nat Commun. 2020;11: 4360. doi:10.1038/s41467-020-18231-z

57. Schroeder ME, McCormack DM, Metzner L, Kang J, Li KX, Yu E, et al. Astrocyte regional specialization is shaped by postnatal development. BioRxiv Prepr Serv Biol. 2024; 2024.10.11.617802. doi:10.1101/2024.10.11.617802

58. Sepp M, Leiss K, Murat F, Okonechnikov K, Joshi P, Leushkin E, et al. Cellular development and evolution of the mammalian cerebellum. Nature. 2024;625: 788–796. doi:10.1038/s41586-023-06884-x
