# Supplementary material for: Cell type-agnostic transcriptomic signatures enable uniform comparisons of neural maturation
Source: PLoS Biol. 2026 Apr 15;24(4):e3003757. doi: 10.1371/journal.pbio.3003757 (PMC13095120; doi:10.1371/journal.pbio.3003757)
Supplement: Attachment — Submitted filename: Response to reviewers_March2026.pdf [file pbio.3003757.s027.pdf]

Dear Dr. Hart,

Thank you for the evaluation of the revisions to our manuscript, *"Cell Type-Agnostic Transcriptomic Signatures Enable Uniform Comparisons of Neurodevelopment,"* at *PLOS Biology*. We are glad that most reviewer comments were answered in our previous revisions. We have now addressed the two additional comments posed by reviewers 1 & 3 below.

Best,

Jesse Gillis, on behalf of all the authors

REVIEWER COMMENTS

Reviewer #1: I very much appreciate the effort by the authors to revise the manuscript and address the concerns from me as well as other reviewers. I think the manuscript has been improved, especially from the biological perspective, that top genes from the cell type-agnostic model now show much more reasonable functions and expression patterns.

I still have a relatively minor concern on this topic though. As the authors reported, the reason why the lowly expressed genes were reported earlier was because coefficients are scaled inversely with gene expression. I assume the authors didn't retrain the model using a different approach in this revised manuscript, just to use a different way to assess feature importance. Since the author used elastic net for regularized regression, and what elastic net regularizes on is the coefficients, does it mean that the unintentionally high coefficients from the lowly expressed genes squeeze the contribution of other genes in the model? Would the model be further improved if the lowly expressed genes are filtered beforehand, or instead of using gene expression as the input, using principal components as a denoised input? For the latter, the PCA loadings can be saved to apply the same transformation to the queue data for model application. This may also be helpful considering the noisy nature of scRNA-seq data.

Reviewer 1 is right that we did not retrain the model, but used two approaches to calculate feature importance: (i) gene usage frequency across bootstrap resampled models (bootstrap score) and (ii) decrease in model performance when the gene's expression is permuted (perturbation score). We appreciate the reviewer's suggestion to evaluate whether removing lowly expressed genes might change the contribution of other genes in the model and improve performance. We retrained models after removing the bottom 5% expressed genes (n=548). This model performs equivalently to the original model: median cross-validation error = 2.7 weeks vs 2.6 weeks originally, Wilcoxon P = 0.95; Spearman R between predicted and actual age = 0.83 vs 0.85 previously). Importantly, the structure of the model remains highly stable after this filtering step. Perturbation-based feature importance scores are nearly identical (R = 0.99) between the two models. Similarly, bootstrap-based gene usage frequencies are largely unchanged: Of 10409 genes now used in model training, 76% show no change, and the remaining genes exhibit only minimal shifts (mean change in frequency: +1.5 / −1.4), with overall usage highly correlated (R = 0.98).

Together, these results indicate that the inclusion of lowly expressed genes does not distort coefficient allocation or suppress the contribution of other genes in the model. Regarding the suggestion to use principal components as input, while this may reduce noise, it would reduce interpretability at the gene level. Our results above suggest that the current gene-level representation is robust to noise introduced by lowly expressed genes.

Reviewer #3: The authors addressed almost all of my previous comments.
Regarding the large variation in predicted ages in organoids, Fig. 7 is still unclear about how within-cell-type (or within-sample?) variability compares with the fetal variability. Also, the calculation of this dispersion of predicted developmental age (SD) conditioned on observed age should be elaborated, especially about "conditioned on".

We apologize for the lack of clarity in our original description. We measured within-sample variability of predicted developmental ages by computing standard deviation of predicted ages per sample (organoid or fetal tissue). We have added the following text to the main manuscript: “Within-sample variability of predicted developmental ages – computed as standard deviation of predictions per sample – is comparable between organoids and primary fetal samples (mean SD: 1.57 vs 2.08 weeks).”

Reviewer #2: The authors have meaningfully addressed my comments
Reviewer #4: In their revised manuscript, the authors have appropriately addressed all reviewers' comments.

We thank the reviewers for their thoughtful comments that helped us improve the manuscript overall.
