# Supplementary material for: Cell type-agnostic transcriptomic signatures enable uniform comparisons of neural maturation
Source: PLoS Biol. 2026 Apr 15;24(4):e3003757. doi: 10.1371/journal.pbio.3003757 (PMC13095120; doi:10.1371/journal.pbio.3003757)
Supplement: Attachment — Submitted filename: Response_to_editorial_requests.docx [file pbio.3003757.s028.docx]

Dear Dr. Hart,

Thank you for the evaluation of our revised manuscript, *“Cell Type-Agnostic Transcriptomic Signatures Enable Uniform Comparisons of Neurodevelopment,”* at *PLOS Biology*. We are glad that the manuscript is likely to be accepted. We agree with the suggestion to modify the title and have revised it to “*Cell Type-Agnostic Transcriptomic Signatures Enable Uniform Comparisons of Neural Maturation*”. We have also uploaded all the data and code needed to generate the figures mentioned by the editorial team to: https://zenodo.org/records/19337213. No changes have been made to the references.

Best,

Jesse Gillis, on behalf of all the authors

-----------------------------------------------------------------------------------------------------------------------------

**Title:
We are not opposed to your paper's current title, but we propose an alternative formulation for your consideration: we wonder if the title would be improved by substituting the word "neurodevelopment" with "neural maturation"? If you opt to change the title, please change it in the form and in the manuscript document when you submit your revision.

Yes, we agree and have updated the title.

**Data & Code:
Thank you for including data and code on Github. We require a few more things to meet our requirements for data and code provision. First, we require the numerical values as they underly the figure plots in the paper. Please include (either in your online repository, or as a supplementary file S1 Data / "S1_Data.xlsx") the values corresponding to the following figure plots:
2DF
3C(dot plot)F
4C
7ACD
S6ABC
S7
S8
S11(right side)
S12A
S13
S15A(right side)B(right side)
S16
Because Github depositions can be readily changed or deleted, we require that code be made available via a permanent DOI’d copy (e.g. in Zenodo) and that this URL be provided in the manuscript and Data Availability Statement, instead of the Github URL. Please also choose a license for your code.
All data to generate the figures mentioned above and code have been uploaded to zenodo (<https://doi.org/10.5281/zenodo.14908185>) We have mentioned this in the manuscript under the methods section, R and R packages. The github repository has been updated with the MIT license.

Please finalize the Data Statement for publication.
We have updated the Data and code availability statement to: “All original code and data used to generate figures are available at https://github.com/sridevi96/NeuroDevTime and https://doi.org/10.5281/zenodo.14908185.”

**Supplement:
Please only upload one copy of each figure.
Please adopt our standard naming scheme for the supplementary tables, eg name them "S1 Table"; "S2 Table", etc. with the filenames according to this format "S1_Table.xlsx", "S2_Table.csv", etc.
[https://journals.plos.org/plosbiology/s/supporting-information](https://can01.safelinks.protection.outlook.com/?url=https%3A%2F%2Fjournals.plos.org%2Fplosbiology%2Fs%2Fsupporting-information&data=05%7C02%7Csridevi.venkatesan%40mail.utoronto.ca%7C6e422c067e5848c49ddc08de8a49e256%7C78aac2262f034b4d9037b46d56c55210%7C0%7C0%7C639100247124936079%7CUnknown%7CTWFpbGZsb3d8eyJFbXB0eU1hcGkiOnRydWUsIlYiOiIwLjAuMDAwMCIsIlAiOiJXaW4zMiIsIkFOIjoiTWFpbCIsIldUIjoyfQ%3D%3D%7C0%7C%7C%7C&sdata=rLNqnfu21XQZO3K9QvTC9fSMO3MBlPttcVS4C6jl%2Bdo%3D&reserved=0)

We have modified the supplementary table file names and uploaded individual tiff files for each supplemental figure. The supporting information captions are at the end of the manuscript file.

-----------------------------------------------------------------------------------------------------------------------------
